# Deciphering and Targeting the Schwannoma‐Neuron‐Macrophage Crosstalk for the Treatment of Schwannomatosis and Associated Pain

**DOI:** 10.1002/advs.202515597

**Published:** 2026-02-27

**Authors:** Zhenzhen Yin, Limeng Wu, Yanling Zhang, Yao Sun, Grace Y. Lee, Simeng Lu, Xing Gao, John W. Chen, Sonu Subudhi, William Ho, Chao Zhu, Jun Ren, Gino B. Ferraro, Alona Muzikansky, Anat Stemmer‐Rachamimov, Jianren Mao, Scott R. Plotkin, Lei Xu

**Affiliations:** ^1^ Edwin L. Steele Laboratories Department of Radiation Oncology Massachusetts General Hospital and Harvard Medical School Boston Massachusetts USA; ^2^ School of Art and Science Tufts University Medford Massachusetts USA; ^3^ Institute for Innovation in Imaging Center for Systems Biology and Division of Neuroradiology Department of Radiology Massachusetts General Hospital Boston Massachusetts USA; ^4^ Division of Biostatistics Harvard Medical School Massachusetts General Hospital Boston Massachusetts USA; ^5^ Molecular Pathology Division Massachusetts General Hospital Boston Massachusetts USA; ^6^ Center For Translational Pain Research Massachusetts General Hospital Boston Massachusetts USA; ^7^ Department of Neurology and Cancer Center Massachusetts General Hospital Boston Massachusetts USA

**Keywords:** anti‐IL‐6, EGF‐R inhibitor, pain, patient‐derived orthotopic model, Schwannoma‐neuron‐macrophage cross talk, schwannomatosis

## Abstract

Non‐NF2 Schwannomatosis (SWN) is a genetic disorder characterized by multiple non‐malignant schwannomas growing on the spine and peripheral nerves. Patients with SWN overwhelmingly present with intractable chronic pain. There are no FDA‐approved drugs to halt tumor growth or alleviate pain. Research on SWN is hindered by the lack of clinically relevant models. We established patient‐derived SWN cell lines from patients with varying pain levels and developed orthotopic patient‐derived xenograft models that reproduce patients’ pain responses. We further developed a novel dorsal root ganglia (DRG) imaging model for longitudinal intravital imaging of macrophage infiltration into the DRG and sensory neuron pain response. Leveraging these novel models, we found that Schwannomas grown distantly in the peripheral nerve caused an influx of macrophages into the DRG. These macrophages in the DRG caused pain via overproducing IL‐6. Treatment with anti‐IL‐6 antibody reduced pain but had modest efficacy in tumor control. We identified epidermal growth factor receptor (EGFR) signaling as a key driver of schwannoma growth and an escape mechanism from anti‐IL6 treatment. Finally, we found that combining IL‐6 and EGFR blockade effectively controlled pain and tumor growth simultaneously in SWN models. In summary, we elucidated the cellular and molecular crosstalk between schwannoma (HMGB1), neuron (CCL2), and macrophage (IL‐6) in driving pain, and identified the EGF signaling pathway as a driver of SWN tumor progression, thereby uncovering novel therapeutic targets that may improve clinical management of SWN.

## Graphical abstract

We established patient‐derived SWN cell lines and orthotopic PDX models that recapitulate patient pain phenotypes, alongside a novel intravital DRG imaging platform to track macrophage infiltration and neuronal pain responses. Using these models, we define HMGB1–CCL2–IL‐6 signaling crosstalk driving pain and identify EGF signaling as a key regulator of SWN tumor progression and therapeutic vulnerability.

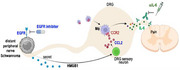

## 1 Introduction

Non‐NF2‐related Schwannomatosis (SWN) is a clinically and genetically distinct form of neurofibromatosis that affects 1 in 126,315 individuals worldwide. The disease is characterized by multiple non‐intradermal schwannomas and a predisposition to other nervous system tumors, including meningiomas. Patients typically present with pain (46%), a mass (27%), or both (11%). Pain is the most frequent symptom reported by patients, with 68% of patients experiencing chronic pain. Schwannomas commonly affect the spine (74%) and peripheral nerves (89%), whereas cranial nerve schwannomas (mostly trigeminal) are uncommon (8%) [1, 2]. SWN is an umbrella term for conditions associated with the genetic predisposition to multiple schwannomas. SWN is primarily associated with germline pathogenic variants in three genes located on chromosome 22q: *SMARCB1*, *LZTR1*, and *NF2* [3, 4]. Germline pathogenic variants in *NF2* are the cause of *NF2*‐related schwannomatosis, previously known as neurofibromatosis 2. Germline pathogenic variants in *SMARCB1* and *LZTR1* account for 70% to 80% of familial non‐*NF2*‐related SWN [5]. Somatic pathogenic variants in *NF2* are frequently present in tumors arising in all forms of SWN [5, 6].

Patients with SWN overwhelmingly present with chronic, intractable pain, which can be severe enough to cause permanent disability [7, 8, 9, 10]. There are no effective therapies that relieve SWN pain—SWN patients with chronic pain are typically treated with medications approved for other medical conditions, evenly split between anti‐inflammatories, neuropathic pain medications, and narcotics. A significant proportion of patients are treated with long‐term narcotics. Despite such aggressive medical treatment with standard medications, a majority of patients rate the pain as moderate‐to‐severe [11, 12]. The etiology of pain in SWN is not clear; the patients describe pain as both neuropathic and nociceptive [9, 13], suggesting that multiple mechanisms may contribute to the pain phenomenon.

Currently, there are no effective therapies that halt SWN tumor growth [13]. Treatment is limited to surgery, which risks iatrogenic nerve injury [11]–neurologic dysfunction related to schwannomas is infrequent, occurring in about 10% of patients at presentation [9]; however, post‐operative neurologic deficits rise to about 45% of patients after spine surgery and 27% of patients after peripheral nerve surgery [9]. The suffering, disability, and lack of FDA‐approved treatments make effective SWN management a significant unmet medical need.

One major obstacle in SWN research is the lack of clinically relevant models. Here, we report the establishment of i) schwannoma cell lines from SWN patients with varying degrees of pain, ii) orthotopic patient‐derived sciatic nerve model and spine model mimicking schwannomas growing on the peripheral nerves and on the spine in patients and reproducing tumor‐induced pain, and iii) a dorsal root ganglia (DRG) imaging model for intravital longitudinal imaging of the DRG microenvironment, which contains the primary sensory neurons and relays pain signals from the peripheral nerve into the central nervous system. Using PDX models, we observed that schwannomas growing on distant peripheral nerves induce a robust influx of macrophages into the DRG. Macrophages are a principal inflammatory cell implicated in inflammatory and neuropathic pain in multiple diseases [14]. These findings led us to hypothesize that crosstalk among the distal Schwannoma, inflammatory macrophages, and DRG neurons contributes to pain. Accordingly, this study was designed with three specific aims: 1) to determine how distally grown schwannomas promote macrophage recruitment into the DRG; 2) to determine how recruited macrophages contribute to pain signaling in the DRG; and 3) to identify the molecular pathways that drive schwannoma tumor growth.

## 2 Results

### 2.1 Establish Orthotopic PDX SWN Models

We established 9 patient‐derived SWN cell lines from surgically resected schwannoma tissues. The indication for surgery in 5/9 patients was pain related to the tumor in question, the pain was spontaneous and breakthrough in 3/5 patients, spontaneous in 1/5 patients, and evoked in 1/5 patients (Table S1, Figure S1A). Because of the non‐malignant nature of schwannomas, among the nine patient‐derived cell lines established, only two form xenograft tumors in mice: SCH‐1 cells from a non‐painful left accessory nerve schwannoma, and SCH‐2 cells from a right pudendal nerve schwannoma with spontaneous and breakthrough pain.

**FIGURE 1:**
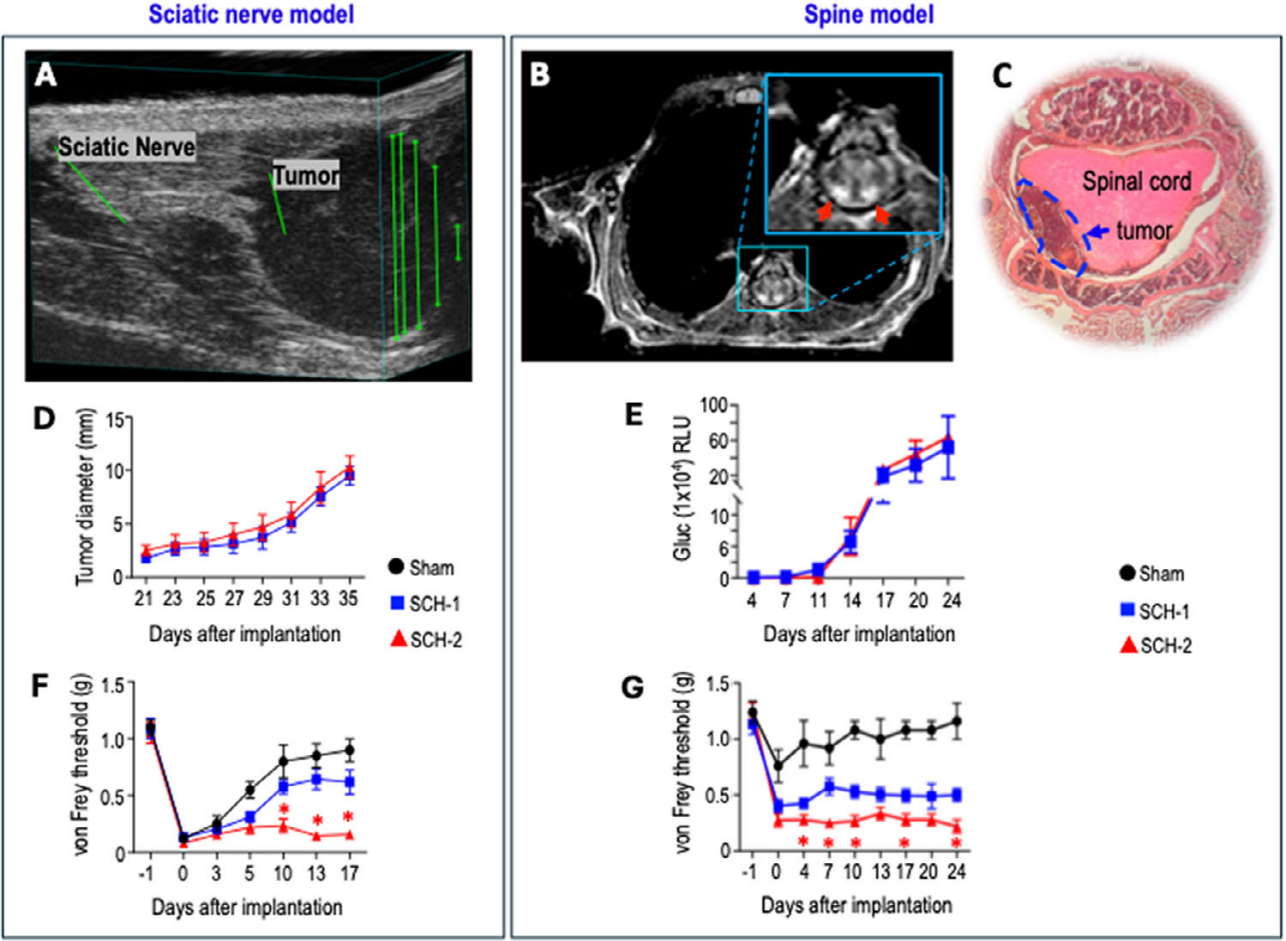
Establish orthotopic PDX SWN models. (A) Sciatic nerve model: 3D ultrasound showed a hypoechoic ovoid mass abutting and exerting mild mass effect. (B) Spine model: post‐contrast MR images of the spine revealed intradural extramedullary masses abutting the posterior spinal cord (red arrows). (C) Spine model: H&E staining showed mild spinal cord compression by the tumor (arrow, circled area). (D) Sciatic nerve model: Tumor growth curve of the SCH‐1 and SCH‐2 models. (E) Spine model: Tumor growth curve of the SCH‐1 and SCH‐2 models. (F) Sciatic nerve model: von Frey filament test in mice receiving Sham surgery, SCH‐1, or SCH‐2 cell implantation. ^*^ Comparing SCH‐1 vs. SCH‐2. Day 10: *p* = 0.001, Day 13: *p* = 0.0002, Day 17: *p* = 0.001. (G) Spine model: von Frey filament test in mice receiving Sham surgery, SCH‐1, or SCH‐2 cell implantation. ^*^ Comparing SCH‐1 vs. SCH‐2. Day 4: *p* = 0.04, Day 7: *p* = 0.004, Day 10: *p* = 0.005, Day 17: *p* = 0.02, Day 24: *p* = 0.01. All animal studies are presented as mean ± SEM, N = 8 mice/group, and are representative of at least three independent experiments. Statistical comparisons between the SCH‐1 vs. SCH‐2 at each time point were analyzed using two‐way ANOVA with post hoc test.

Given that SWNs grow on peripheral nerves (89% of patients) and the spine (74% of patients) [1, 9], we implanted SWN cells orthotopically in the sciatic nerve and spine. In the sciatic nerve model, 3D ultrasound confirmed tumor formation and location relative to the sciatic nerve (Figure 1A). In the spine model, MR imaging and H&E staining confirmed that schwannoma xenografts grew in the intradural and extramedullary space of the spine (Figure 1B,C), reproducing the correct anatomical location of spinal schwannomas in patients. In both the sciatic nerve and spine models, tumors formed exclusively at the injection site and were not observed to extend into nerves or other anatomical locations. SCH‐1 and SCH‐2 tumors grew at similar rates (Figure 1D,E). Histological staining for S100 and Sox10 confirmed their Schwann cell origin and schwannoma histology (Figure S1B,C).

Pain behavior was evaluated using the von Frey filament test for mechanical allodynia and the spontaneous lift and licking tests for non‐evoked pain. In the sciatic nerve model, Sham surgery induced mechanical allodynia and spontaneous lifting of the ipsilateral hind paw, which resolved within 5–10 days post‐surgery (Figure 1F; Figure S1D). No spontaneous licking was observed in the Sham group (Figure S1E). In the tumor implantation group, we observed sustained mechanical allodynia post‐surgery (Figure 1F). The spontaneous lift and licking tests did not detect pain after mice recovered from surgery, around day 7 (Figure S1D,E), therefore, we used the mechanical allodynia test to evaluate pain in our studies. In the spine model, as intrathecal injection does not require surgery, Sham injection only caused a mild pain response that returned to the normal level within 3 days. Tumor growth caused persistent mechanical allodynia (Figure 1G). In both sciatic nerve and spine models, although grown at a similar rate, the **painful SCH‐2** tumors induced significantly greater pain responses than the non‐painful SCH‐1 tumors (Figure 1F,G).

Mice bearing SCH‐1 or SCH‐2 tumors had comparable motor functions as evaluated by rotarod assay (Figure S1F), and we observed no changes in the motor function up to 3 weeks post‐implantation (Figure S1G,H). Additionally, both male and female mice showed comparable tumor‐induced pain responses (Figure S1I).

### 2.2 Schwannomas Exhibit an Inflammatory Signature Characterized by Elevated Macrophage Infiltration

**FIGURE 2:**
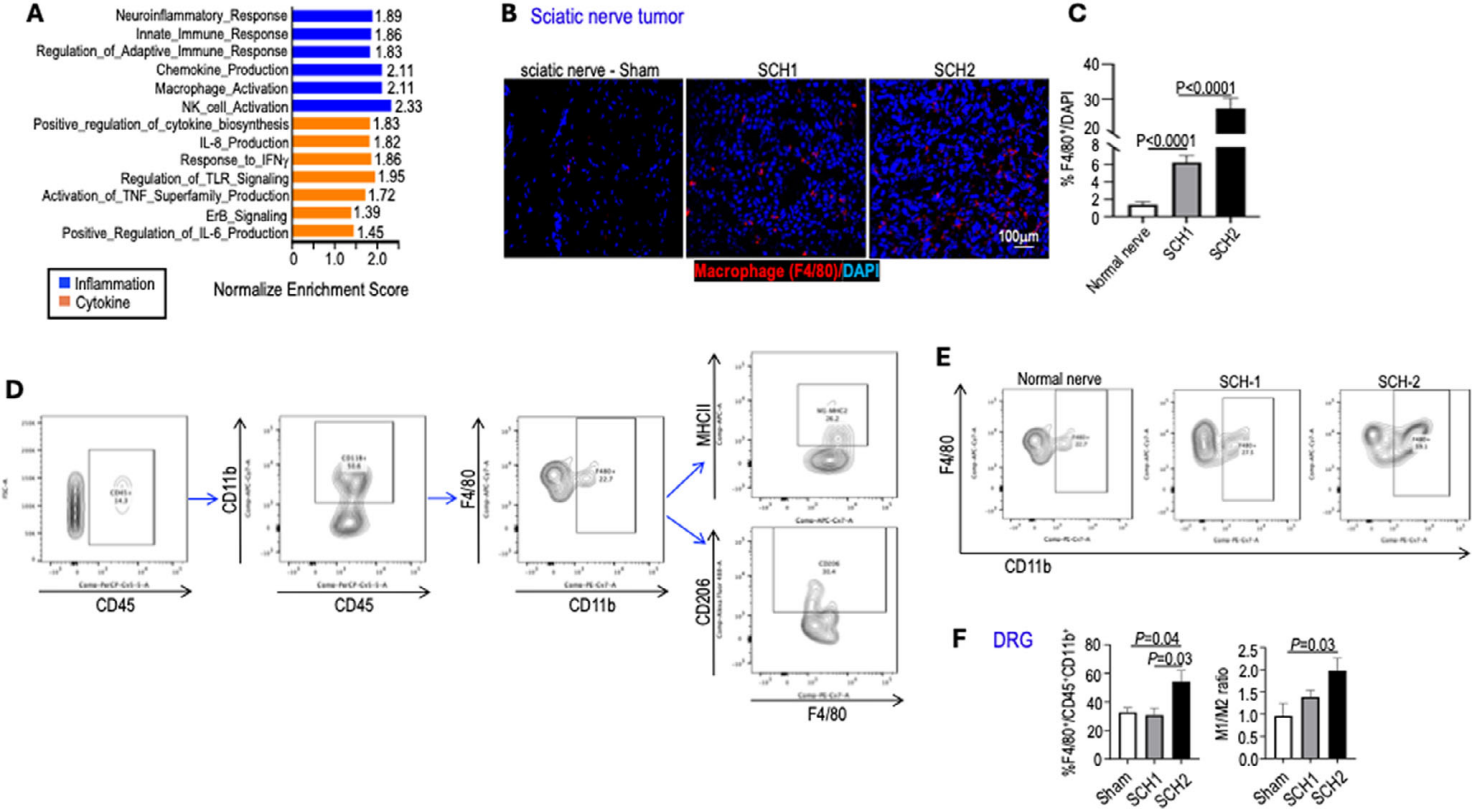
Schwannomas exhibit an inflammatory signature characterized by elevated macrophage infiltration. (A) Normalized enrichment scores from RNASeq analysis of patient schwannomas (SCH‐1, SCH‐2, and SCH‐4). (B) Representative images of IF staining for macrophages (F4/80, red) in the sciatic nerve tumor. The sciatic nerves collected from the Sham group were included as controls. DAPI, blue. (C) The percentage of macrophage (F4/80^+^/DAPI) was manually counted in 10 randomly selected fields per tumor, across 3 sciatic nerve tumors per group. N = 30 fields/group. (D) Flow cytometry gating strategy for DRG immune cell analysis. Representative gating is shown for singlets, live cells, and CD45^+^ immune cells from dissociated DRGs. Macrophages were gated as CD11b^+^F4/80^+^ within the CD45^+^CD11b^+^ population, and further analyzed for M1 (MHCII^+^) and M2 (CD206^+^) markers. Data are presented as percentages of CD45^+^CD11b^+^ cells. (E) Representative gating for DRG macrophages in the Sham, SCH‐1, and SCH‐2 groups. (F) Flow cytometry analysis of the number of macrophages in lumbar DRGs (L3‐L6, N = 4 DRGs/mice) ipsilateral to sciatic nerve tumor. The ratio of M1‐like macrophage (MHCII^+^F4/80^+^CD11b^+^CD45^+^) to M2‐like macrophage (CD206^+^F4/80^+^CD11b^+^CD45^+^) was calculated. 3 mice/group. N = 12 DRGs/arm. Image quantification and flow cytometry data are presented as mean ± SD. Statistical comparisons were analyzed using an unpaired two‐tailed Student's t‐test. ^*^ *P* < 0.05.

To decipher the mechanism of tumor‐induced pain response, we compared the transcriptomes of patient schwannomas with those of normal Schwann cells (NSC) using bulk RNA sequencing (RNASeq). RNA was extracted from freshly resected patient schwannomas (SCH‐1, SCH‐2, and SCH‐4–another painful left tibial nerve schwannoma), and from normal Schwann cells obtained from the great auricular nerve. Pathway enrichment analysis on 487 differentially expressed genes (FDR q‐value<0.05 and Log2FC>2, Table S2) revealed that elevated genes in schwannomas are enriched in pathways related to: i) neuroinflammatory response, ii) inflammatory cell activation, such as macrophages and NK cells, and iii) production of inflammatory cytokines (TNF‐α, IL‐6, and IFN‐γ) (Figure 2A).

Consistent with this inflammatory gene signature, mice bearing SCH‐1 or SCH‐2 tumors exhibited more macrophage infiltration in sciatic nerve tumors compared to the non‐tumor‐bearing Sham group (Figure 2B,C). In addition to nociceptive signaling generated at the tumor site, macrophages in the nervous system contribute to pain initiation and persistence [15, 16, 17]. We therefore examined macrophage infiltration in the brain, spinal cord, and DRG using flow cytometry (Figure 2D,E). In the sciatic nerve model, compared with Sham‐injected controls, both SCH‐1 and SCH‐2 tumor growth increased macrophage numbers in the spinal cord and brain; however, these differences did not reach statistical significance at either site (Figure S2A,B). Compared to both Sham controls and the non‐painful SCH‐1 group, mice bearing painful SCH‐2 tumors exhibited a significant increase in macrophage infiltration in the ipsilateral DRGs (L3‐6), and a marked shift toward the pro‐inflammatory M1 phenotype (Figure 2F). To determine whether this response was localized to the tumor‐bearing side, we examined contralateral DRGs in the SCH‐2 sciatic nerve model. Macrophage levels in contralateral DRGs (L3‐6) were comparable to those in Sham controls and were significantly lower than levels in the ipsilateral DRGs (Figure S2C).

### 2.3 Peripheral Nerve Schwannoma Triggers Macrophage Influx into the DRG

**FIGURE 3:**
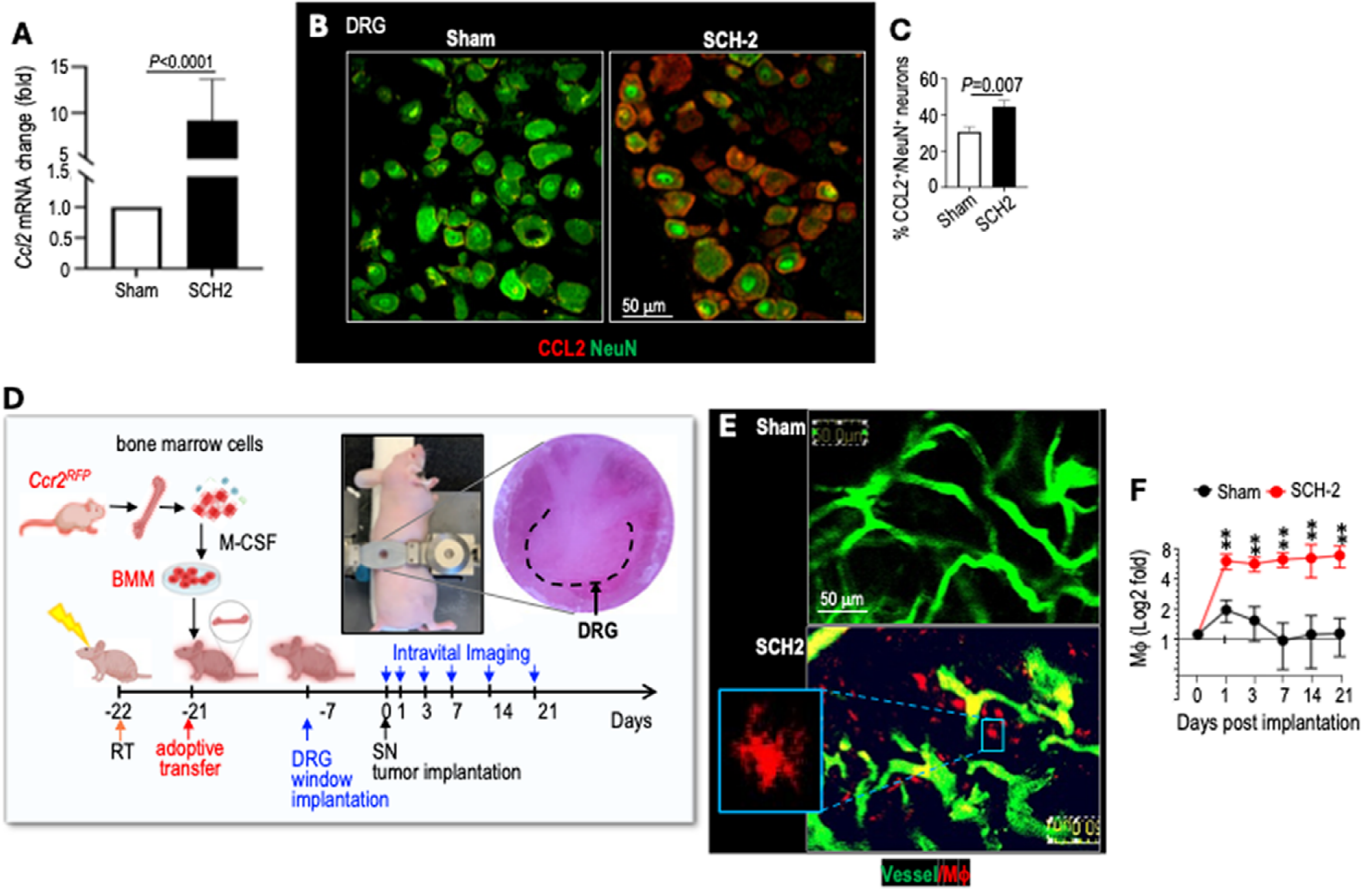
Peripheral nerve schwannomas trigger macrophage influx into the DRG. (A) Mouse *Ccl2* mRNA expression level was evaluated in the lumbar DRGs (L3‐L6) from the Sham or SCH‐2 group by qRT‐PCR. N = 3 tumors/group, tested in triplicate. (B) Representative images of IF staining of CCL2 (red) in neurons (NeuN, green) of ipsilateral lumbar DRGs (L3‐L6, N=4 DRGs/mice, N=3 mice/group) in the sciatic nerve model. (C) The percentage of CCL2‐expressing neurons (CCL2^+^/NeuN^+^) was manually counted in 10 randomly selected fields (N = 120 fields/group). (D) Schematic and timeline of intravital DRG imaging of adoptively transferred BMM in the sciatic nerve model. (E) 2‐photon images of blood vessels (green) and infiltrating *Ccr2^RFP^* macrophages (red) in the DRG on day 14 post‐implantation (N = 4 mice/group). (F) Red fluorescent macrophages were counted in 5 random fields per mouse (N = 20 fields/group). Data presented as mean ± SD. Statistical comparisons were analyzed using unpaired two‐tailed Student's t‐test. ^*^ *P* < 0.05, ^**^ *P* < 0.005.

Next, we investigated how a distant peripheral nerve tumor causes macrophage influx into the DRG. C‐C motif ligand 2 (CCL2) is a chemokine that recruits macrophages [18]. In mice bearing SCH2 tumors, we observed elevated *Ccl2* mRNA (Figure 3A) and protein levels in DRG sensory neurons (Figure 3B,C).

To dynamically characterize how tumor growth affects macrophage infiltration into the DRG, we custom‐designed and 3D‐printed a DRG imaging window and surgically implanted it over the DRG (Figure 3D). We visualized macrophages by adoptively transferring bone marrow‐derived macrophages (BMM) from C‐C chemokine receptor 2 (CCR2) reporter mice *(Ccr2^RFP^)*. Since CCR2 is primarily expressed on macrophages and is the main receptor for CCL2 [18], the red fluorescent protein expression in macrophages allowed us to track macrophages at the tissue site. However, the reporter mice are in the immune‐competent C57BL/6 background. To remedy this and grow the patient‐derived tumors, we transferred BMM from *Ccr2^RFP^* mice into whole‐body irradiated nude mice. After successful generation of bone marrow chimeras, we implanted the imaging window over the DRG. We waited seven days to minimize inflammation and pain from the window implantation before implanting the tumor in the sciatic nerve. We performed intravital 2‐photon imaging in live animals to capture the dynamic trafficking of macrophages from the blood vessel into the DRG. In this imaging approach, only infiltrating *Ccr2^RFP^* macrophages are fluorescently labeled, whereas resident DRG macrophages, which do not express RFP, are not visualized. Intravital imaging of the DRG showed a 5.3 ± 0.92‐fold increase in red fluorescent *Ccr2^RFP^* macrophages one day after tumor implantation. This increase persisted until day 21 post‐tumor implantation, while non‐tumor‐bearing mice exhibited few to no RFP‐expressing macrophages in the lumbar DRGs (Figure 3E,F).

### 2.4 Macrophages in the DRG are Essential in Schwannoma‐Induced Pain

**FIGURE 4:**
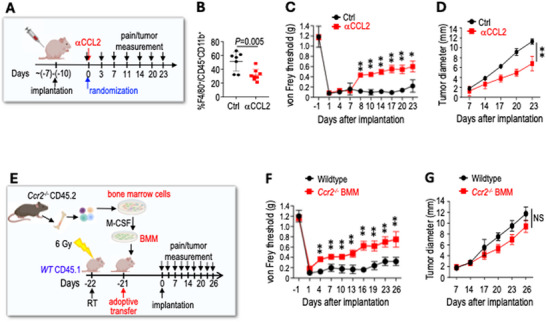
Macrophage in the DRG is essential in schwannoma‐induced pain. (A) Schematic and timeline of anti‐CCL2 antibody (aCCL2) treatment in the sciatic nerve model. (B) Flow cytometry of macrophages in lumbar DRGs ipsilateral to sciatic nerve tumor on day 23 post‐implantation (Ctrl: N = 7, a CCL2: N = 8 DRGs). (C) von Frey filament tests. (D) Tumor diameter measured by caliper. (E) Schematic and timeline of blocking macrophage infiltration by adoptive transfer of BMM from Ccr−/− mice. (F) von Frey filament tests. (G) Tumor diameter measured by caliper. All animal studies presented are mean ± SEM, N = 8 mice/group, and represent at least three independent experiments. Statistical comparisons of tumor growth were performed by two‐way repeated measures ANOVA, and pain responses at each individual time point were analyzed using ANOVA with a post hoc test. Flow cytometry data are presented as mean ± SD and analyzed using unpaired two‐tailed Student's t‐test. *P < 0.05, **P < 0.005.

To examine the functional role of macrophages in SWN‐induced pain, we blocked macrophage infiltration using two strategies. First, we treated mice with an anti‐CCL2 antibody (Figure 4A). In the SCH‐2 model, characterized by high macrophage infiltration, the CCL2 antibody treatment: i) reduced macrophage infiltration into the DRG (Figure 4B, ii) decreased mechanical allodynia (Figure 4C), and iii) delayed tumor growth (Figure 4D). In contrast, in the non‐painful SCH‐1 model with fewer macrophages in the DRG, CCL2 antibody treatment did not further reduce macrophage numbers, nor did it affect mechanical allodynia or tumor growth (Figure S3A–C). Previous studies have shown that CCL2 may directly cause central sensitization in chronic pain models [19, 20, 21]. To differentiate macrophage‐induced inflammatory pain from the potential direct effects of CCL2 on neurons, we blocked macrophage infiltration by adoptive transfer of bone marrow‐derived macrophages from *Ccr2^−/−^* mice (Figure 4E). Since CCR2 is required for 80%–90% of macrophage recruitment and *Ccr2^−/−^* mice exhibit defective macrophage chemotaxis [22], we observed fewer macrophages in the DRG in the chimera mice (Figure S3D). Compared with wild‐type mice, chimera mice exhibited reduced allodynia (Figure 4F) while maintaining the same tumor growth rate (Figure 4G). These studies reveal that macrophages are essential in mediating tumor‐induced pain in SWN models.

### 2.5 Schwannoma‐Secreted HMGB1 Upregulates Neuronal CCL2 Expression to Recruit Macrophages into the DRG

**FIGURE 5:**
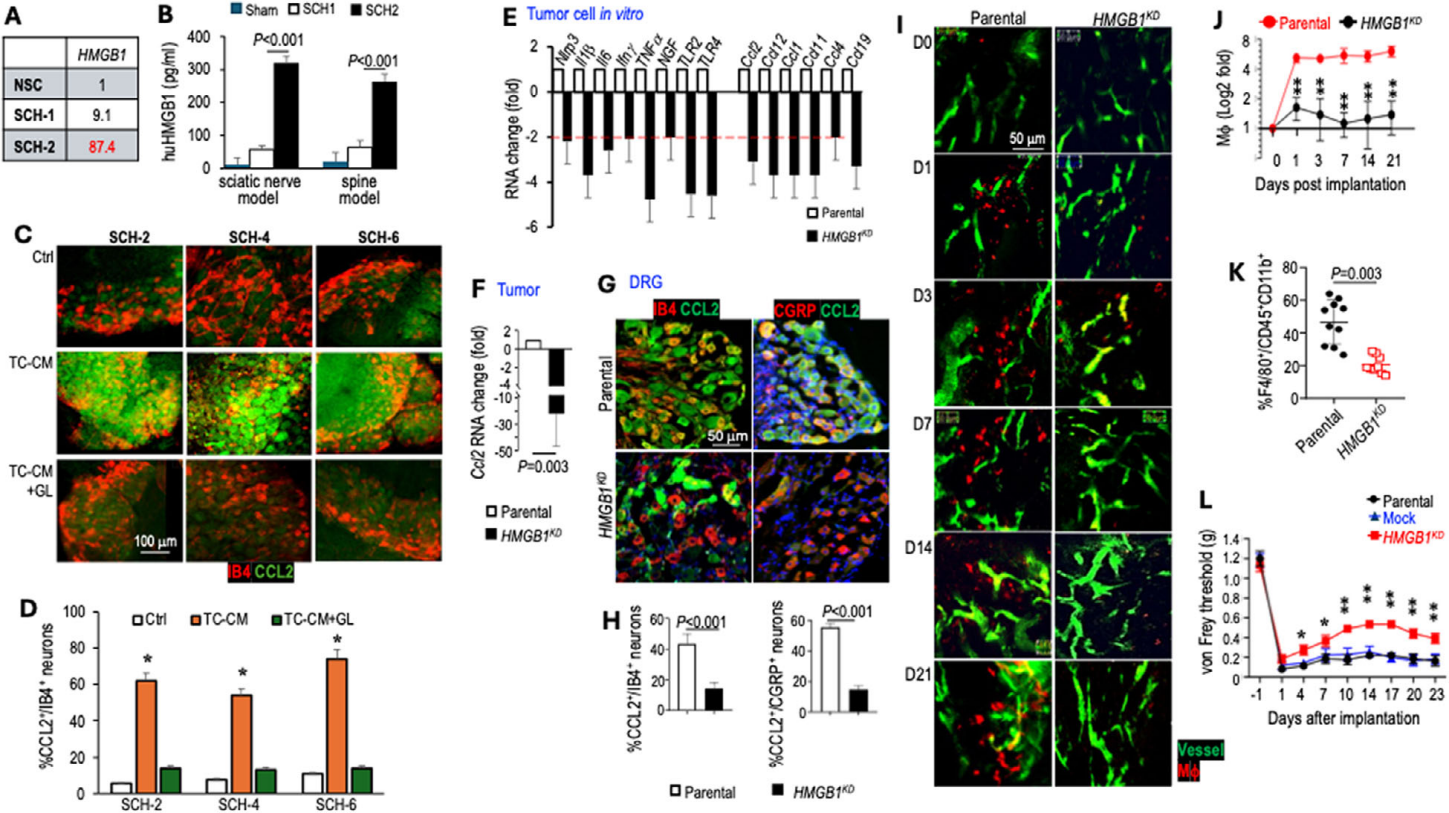
Schwannoma‐secreted HMGB1 upregulates neuronal CCL2 expression to recruit macrophages into the DRG. (A) qRT‐PCR of HMGB1 in normal Schwann cell (NSC) and SCH‐1 and SCH‐2 cells. (B) ELISA of human HMGB1 in plasma from sciatic nerve and spine mouse models (N=4 tumors/group, tested in triplicate). (C) DRGs organotypically cultured in DMEM (Ctrl), SCH‐2, SCH‐4, and SCH‐6 tumor cell conditioned medium (TC‐CM), or TC‐CM+HMGB1 inhibitor, Glycyrrhizin (GL, 50 µg/ml) for 48 h. Representative image of IF staining for CCL2 (green) in small‐diameter (IB4^+^, red) neurons. (D) Image quantification of the percentage of CCL2 expressing small neurons (IB4^+^) in 10 randomly selected fields (N=30 fields/group). (E) qRT‐PCR analysis of inflammatory cytokine and chemokine gene expression of parental and HMGB1^KD^ cells. (F) qRT‐PCR analysis of mouse *Ccl2* mRNA in parental or HMGB1^KD^ sciatic nerve tumors (N = 3 tumors/group, tested in triplicate). (G) IF staining of CCL2 (green) in small (IB4^+^, red) and large (CGRP^+^, red) neurons in lumbar DRGs (L3‐6, N=4/mouse) ipsilateral to the parental or HMGB1^KD^ sciatic nerve tumors. N=3 mice/group. (H) Image quantification of the percentage of CCL2 expressing small (CCL2^+^/IB4^+^) and large (CCL2^+^/CGRP^+^) neurons in 10 randomly selected fields (N = 120 fields/group). (I) 2‐photon imaging of macrophage (red) infiltrating the DRG at different time points post‐tumor implantation. DRG vessels (green). N = 4 mice/group. (J) Fold change of macrophages in parental vs. *HMGB1^KD^* tumors, manually counted in five random fields per mouse (N = 20 fields/group). (K) Flow cytometry analysis of macrophages in lumbar DRGs (L3‐L6, N=4 DRGs/mice from 3 mice/group) ipsilateral to sciatic nerve tumor implantation (N = 12 DRGs/group). (L) von Frey filament tests in parental and *HMGB1^KD^* sciatic nerve models. Data presented are mean ± SEM, N = 8 mice/group, and represent at least three independent experiments. Image quantification, qRT‐PCR, and flow cytometry data are presented as mean ± SD. Statistical comparisons were analyzed using unpaired two‐tailed Student's t‐test. Pain responses at each individual time point were analyzed using ANOVA with post hoc test. ^*^ *P* < 0.05, ^**^ *P* < 0.005.

To identify the driver of macrophage recruitment, we examined transcriptome profiling data from the Figure 2A study and found that both SCH‐1 and SCH‐2 tumors express higher levels of HMGB1 than normal Schwann cells (Figure 5A). HMGB1, a potent inflammation initiator and amplifier, has been shown to upregulate CCL2 in smooth muscle cells in an artery injury model [23]. We confirmed that patient Schwannoma cells secrete HMGB1 in vitro (Figure S4A). In both the sciatic nerve and spine models, secreted HMGB1 was detected in blood samples from mice bearing SCH‐1 and SCH‐2 tumors. Notably, HMGB1 levels were significantly elevated in the SCH‐2 model, both in non‐tumor‐bearing Sham controls and in non‐painful SCH‐1 models (Figure 5B).

To determine whether HMGB1 regulates neuronal CCL2 expression, we employed an ex vivo DRG organotypic culture model. Tumor cell‐conditioned medium (TC‐CM) from painful SCH‐2, SCH‐4, and SCH‐6 cell lines robustly induced CCL2 expression in DRG neurons. Pharmacologic inhibition of HMGB1 with glycyrrhizin (GL) effectively abolished this tumor‐induced CCL2 upregulation in both small‐diameter IB4^+^ neurons (Figure 5C,D) and large‐diameter CGRP^+^ neurons (Figure 4B,C). Together, these findings demonstrate that HMGB1 is a key mediator of tumor‐induced neuronal CCL2 expression.

To confirm HMGB1's direct causal role in neuronal CCL2 upregulation, we used CRISPR‐Cas9 gene editing to genetically silence HMGB1 expression in SCH‐2 cells, which express high levels of HMGB1. Three single‐cell clones with the lowest HMGB1 levels were picked and pooled to avoid clonal variation (Figure S4D). HMGB1 knockdown significantly downregulates multiple inflammatory mediators, including ***Nlrp3, Il‐1β, Il‐6***, ***Ifn‐γ***, *Tnf‐α*, *Ngf*, *Tlr2*, and *Tlr4*. In addition, we observed decreased expression of chemokines that recruit monocyte/macrophages (***Ccl2*** and ***Ccl12)***, T cells ***(Ccl1 *** and ***Ccl11)***, dendritic cells (***Ccl19***), and NK cells (***Ccl4***) (Figure 5E). HMGB1 knockdown did not affect tumor cell viability in vitro (Figure S4E). In the sciatic nerve model, we confirmed that HMGB1 knockdown reduced CCL2 expression in tumors (Figure 5F) and DRG sensory neurons (Figure 5G,H), but did not affect tumor growth in the PDX model (Figure S4F).

To investigate HMGB1's functional role in macrophage recruitment, we used the DRG imaging model for intravital imaging of macrophage infiltration in live animals. In the same experiment setting as described in Figure 3D study, we transferred bone marrow‐derived macrophages from *Ccr2^RFP^* reporter mice into whole‐body irradiated nude mice. After generating bone marrow chimeras, we implanted the imaging window over the DRG. Seven days later, we implanted either parental or HMGB1‐knockdown tumor cells into the sciatic nerve and used multiphoton microscopy to detect red‐fluorescent macrophages in the DRG at different time points. In mice bearing HMGB1‐knockdown tumors, fewer macrophages were observed in the DRG throughout the course of tumor growth (Figure 5I,J). Flow cytometry quantitatively confirmed that HMGB1 knockdown reduced macrophage numbers in the lumbar DRGs (Figure 5K). Consistent with reduced macrophage infiltration, mice bearing HMGB1 knockdown tumors exhibited reduced mechanical allodynia (Figure 5L).

### 2.6 αIL6 Treatment Reduces Pain

**FIGURE 6:**
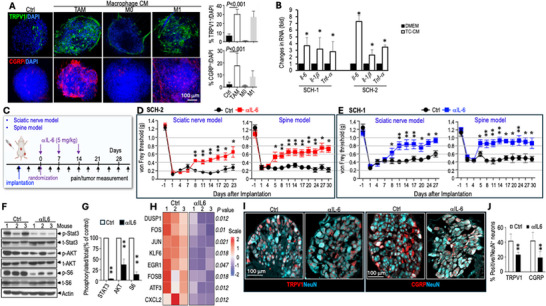
αIL6 treatment reduces pain. (A) Representative images of IF staining and image quantification for TRPV1 (green) and CGRP (red) in organotypic DRGs ex vivo culture (N = 3 DRGs/group). DAPI, blue. The percentage of TRPV1 and CGRP expressing neurons were manually counted in 10 randomly selected fields (N=30 fields/group). (B) qPCR analysis of murine *Il‐6*, *Il‐1β*, and *Tnf‐α* in bone marrow‐derived macrophages cultured in DMEM and tumor cell conditioned medium. (C) Schematic and timeline of αIL‐6 treatment experiment. (D) von Frey filament tests in SCH‐2 sciatic nerve and spine models. (E) von Frey filament tests in SCH‐1 sciatic nerve and spine models. (F) Western blot of tumor tissues (N=3 tumors/group). (G) Densitometry of western blot. (H) Heatmap of single‐cell RNAseq analysis of TAMs isolated from control and αIL6‐treated tumors (N=3 tumors/group). (I) Representative images of IF staining for TRPV1 (red) and CGRP (red) in neurons (NeuN^+^, blue) in DRG (L3‐6, N=4 DRGs/mice) ipsilateral to sciatic nerve tumor. N=3 mice/group. (J) The percentage of TRPV1 and CGRP expressing neurons were manually counted in 10 randomly selected fields (N = 120 fields/group). All animal studies presented are mean±SEM, N = 8 mice/group, and represent at least three independent experiments. Pain responses at each individual time point were analyzed using ANOVA with post hoc test. Image quantification, qRT‐PCR and densitometry data are presented as mean ± SD. Statistical comparisons were analyzed using unpaired two‐tailed Student's t‐test. ^*^ *P* < 0.05, ^**^ *P* < 0.005.

To investigate how SWN triggers macrophage inflammatory responses to induce pain, we differentiated Raw264.7 macrophages to different phenotypes by treating them with: i) tumor cell‐conditioned medium (TC‐CM)—representing TAMs, ii) regular culture medium—representing naïve M0 phenotype, and iii) IFN‐γ (1000 U/ml)—inducing an M1 inflammatory phenotype, serving as a positive control for nociception‐related signaling [24, 25]. We collected CM from these macrophage cultures and added them to the organotypic DRG ex vivo culture. After 3 days, CM from TAMs and M1 inflammatory macrophages induced TRPV1 and CGRP expression in DRG neurons (Figure 6A).

To investigate how SWN tumor cells affect macrophage inflammatory responses, we compared the effects of TC‐CM from the non‐painful (SCH‐1) and painful (SCH‐2) cells on cytokine expression in bone marrow‐derived macrophages (BMDMs), which more closely reflect physiological macrophage responses. TC‐CM from both cell lines stimulated the expression of proinflammatory cytokines, including *Il‐6*, *Il‐1β*, and *Tnf‐α*, with IL‐6 exhibiting the highest induction (Figure 6B).

Given that IL‐6 is a major trigger of inflammatory pain [26, 27, 28], and that anti‐IL‐6 and anti‐IL‐6 receptor antibodies are FDA‐approved for pain management [29, 30], we focused on testing IL‐6 blockade (Figure 6C). In both SCH‐1 and SCH‐2 models, anti‐IL‐6 (αIL‐6) neutralizing antibody alleviated pain in both sciatic nerve and spine models (Figure 6D,E). αIL‐6 treatment modestly delayed tumor growth in both SCH‐2 and SCH‐1 models (Figure S5A,B). At the molecular level, we found that αIL‐6 treatment i) reduced inflammatory STAT3 and AKT/S6 phosphorylation in sciatic nerve tumors by western blot analysis (Figure 6F,G), ii) reduced inflammatory gene expression in TAMs by single‐cell RNASeq (Figure 6H; Table S2), and iii) reduced TRPV1 and CGRP expression in DRG sensory neurons by IF staining (Figure 6I,J).

### 2.7 Combined IL‐6 and EGFR Blockade Concurrently Controls Tumor Growth and Pain

**FIGURE 7:**
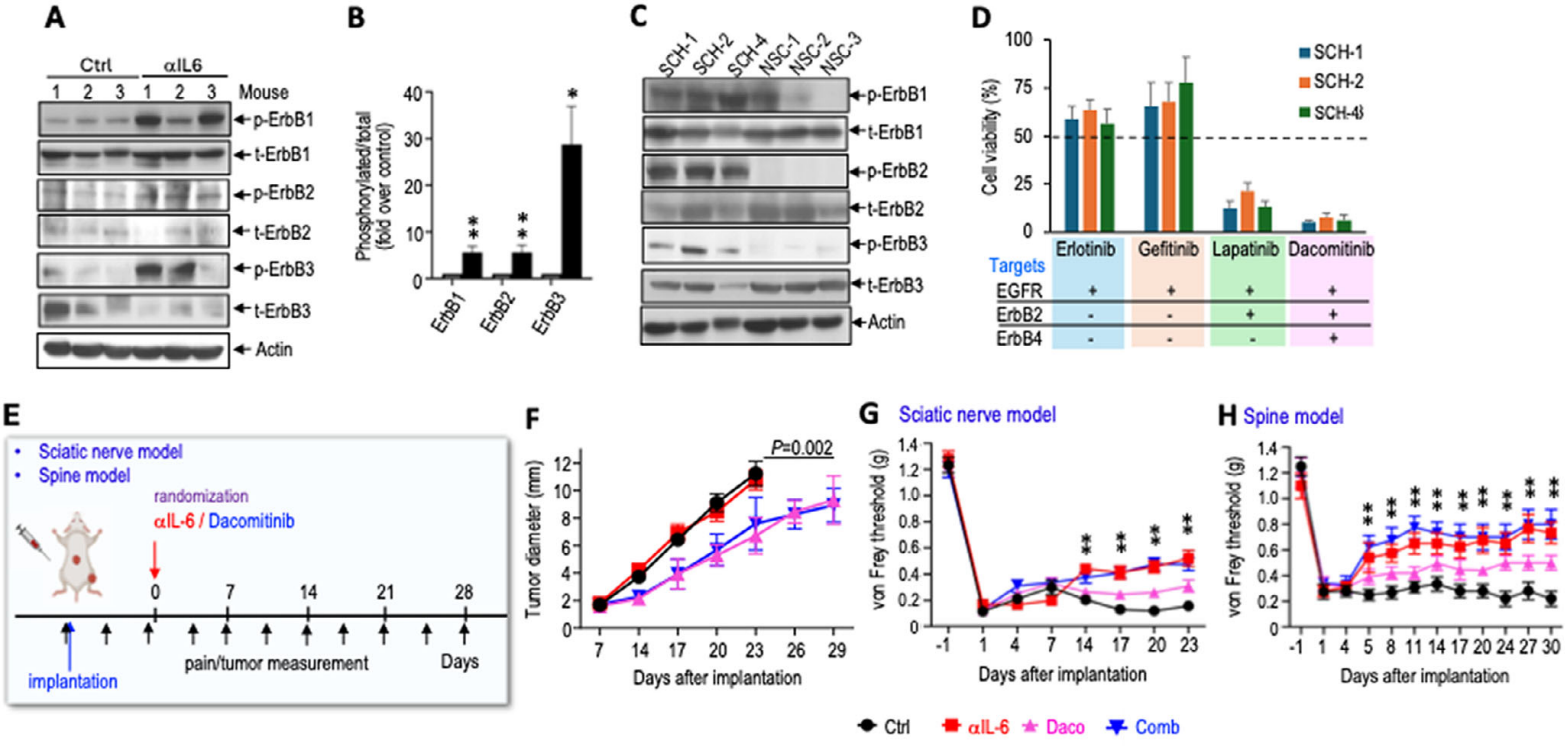
Combined IL‐6 and EGFR blockade concurrently control tumor growth and pain. (A) Western blot of tumor tissues for EGFR family receptors. (B) Densitometry of western blot. (C) Western blot of patient‐derived SCH‐1, ‐2, ‐4 cells, and normal Schwann cells (NSC) for EGFR family receptors. (D) In vitro drug screening by MTT assay. SCH‐1, ‐2, ‐4 cells were treated with DMEM or 10 µm of each EGFR inhibitor for 72 h. (E) Schematic and timeline of αIL‐6 and dacomitinib treatment experiment. (F) Sciatic nerve SCH‐2 tumor size was measured by caliper. (G) von Frey filament test in the SCH‐2 sciatic nerve model. (H) von Frey filament test in the SCH‐2 spine model. All animal studies presented are mean ± SEM, N = 8 mice/group, and represent at least three independent experiments. Statistical comparisons of tumor growth were performed by two‐way repeated measures ANOVA, pain responses at each individual time point were analyzed using ANOVA with post hoc test. Densitometry and MTT data are presented as mean ± SD and analyzed using unpaired two‐tailed Student's t‐test. ^*^ *P* < 0.05, ^**^ *P* < 0.005.

In sciatic nerve tumors, αIL‐6 treatment induced phosphorylation of EGFR and ErbB2/3, indicating activation of EGF signaling (Figure 7A,B). Since EGFR signaling promotes tumor progression [31, 32], this suggests a potential escape mechanism that could explain the low efficacy of tumor control with αIL‐6 treatment. Patient‐derived schwannoma SCH‐1, SCH‐2, and SCH‐4 cells showed elevated baseline phosphorylation of EGFR, ErbB2, and ErbB3 compared to normal Schwann cells (Figure 7C), suggesting EGF signaling may be a driver of schwannoma progression.

To test the efficacy of EGFR blockade, we screened four FDA‐approved EGFR tyrosine kinase inhibitors (TKIs) in vitro [33]. Among them, lapatinib (an EGFR and HER2 TKI) and dacomitinib (a pan‐ErbB inhibitor) reduced the viability of patient‐derived SCH‐1, SCH‐2, and SCH‐4 schwannoma cells, while erlotinib and gefitinib (EGFR inhibitors) had minimal effects (Figure 7D). We focused on testing dacomitinib efficacy in vivo (Figure 7E), which showed the most effective inhibition of cell growth in vitro.

In the PDX model, dacomitinib treatment alone or in combination with αIL‐6 delayed tumor growth compared with control or αIL‐6 alone. However, the combination did not exhibit additive effects beyond those of dacomitinib alone (Figure 7F). In both sciatic nerve and spine models, dacomitinib monotherapy minimally reduced pain, while αIL‐6 alone or in combination with dacomitinib reduced pain without additive effects (Figure 7G,H). Dacomitinib monotherapy reduced tumor cell proliferation (PCNA^+^) and increased tumor cell apoptosis (TUNEL^+^), but the combination with αIL‐6 did not enhance these effects (Figure S5C). None of the treatments caused systemic toxicity or affected motor function in mice (Figure S5D,E).

### 2.8 IL‐6 and EGFR are Valid Targets in Patients with SWN

**FIGURE 8:**
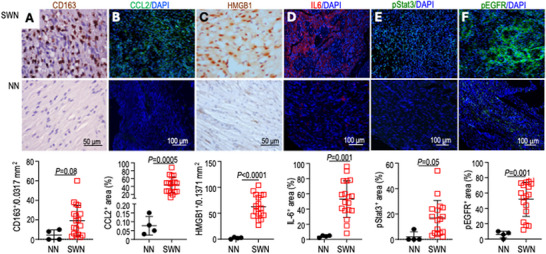
IL‐6 and EGFR are valid targets in patients with SWN. Archived paraffin‐embedded tumors from patients with SWN (n=18) were immunostained for (A) macrophage (CD163^+^, brown), (B) CCL2 (green), (C) HMGB1 (brown), (D) IL‐6 (red), (E) phosphorylated STAT3 (green), and (F) phosphorylated EGFR (green). NN: normal nerve (N = 4). The number of TAMs and HMGB1 positively stained cells was counted manually in 20 random fields (0.0317 mm^2^ field). Fluorescent images were analyzed for the % positive stained area using ImageJ software. Statistical comparisons between the groups was analyzed using a Student's t‐test.

To prepare the translation of our study into the clinic, we validated our preclinical results using archived human SWN tumor samples. Compared with the normal nerve, we observed a trend toward increased infiltrating TAMs (CD163^+^), and significantly elevated CCL2, HMGB1, and IL‐6 expression, as well as phosphorylation of STAT3 and EGFR (Figure 8A–F). These results confirm that IL‐6 and the EGFR are valid targets for SWN.

## 3 Discussion

The drivers of pain response and tumor progression in SWN remain unclear, and there are no FDA‐approved drugs to halt schwannoma growth or alleviate SWN‐associated pain. Schwannomatosis research is limited by the lack of clinically relevant models. Here, we report the establishment of patient‐derived cell lines from SWN patients with varying degrees of pain, two orthotopic PDX models that reproduce tumor‐induced pain, and a DRG imaging model that allows real‐time, longitudinal imaging of the DRG microenvironment in live animals.

**FIGURE 9:**
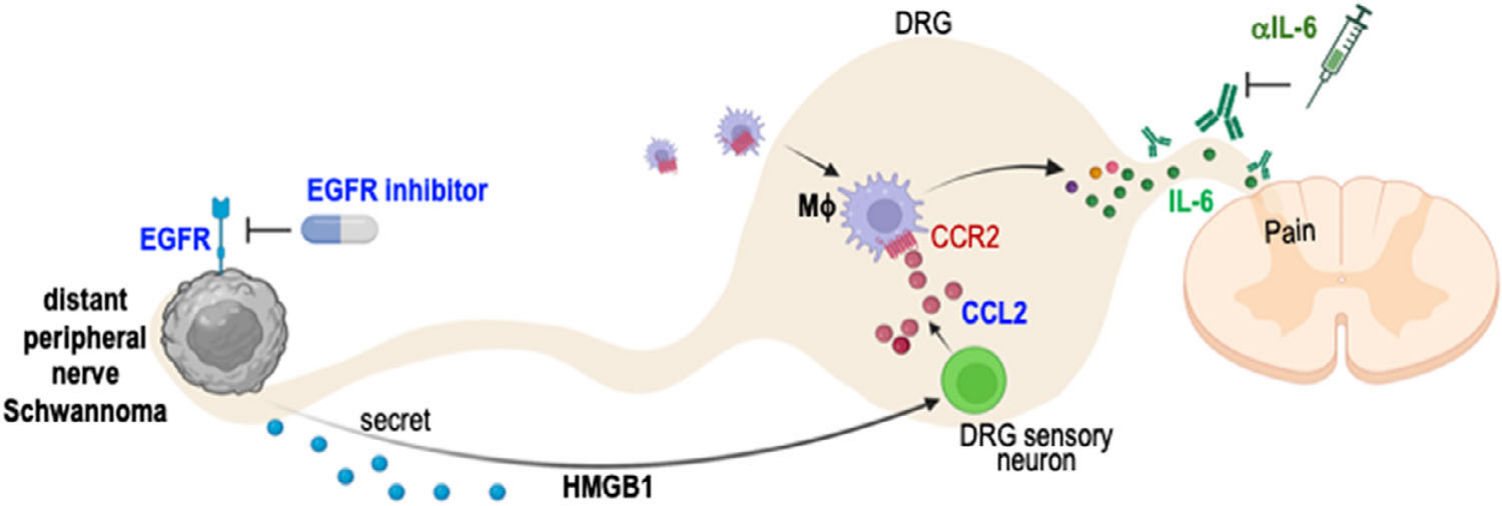
Graphic summary of the study findings.

Leveraging these innovative tools, we deciphered the cellular and molecular crosstalk between schwannoma–neuron—macrophage that drives pain. We demonstrated that i) Schwannomas growing distally in the peripheral nerve secrete HMGB1, which enters the circulation; ii) circulating HMGB1 stimulates sensory neurons in the DRG to express CCL2; iii) CCL2 then recruits CCR2^+^ macrophages into the DRG; and iv) these macrophages contribute to pain through elevated production of IL‐6. Blocking IL‐6 reduced pain but had modest efficacy in tumor control. We identified EGF signaling as a driver of schwannoma growth and an escape mechanism from anti‐IL6 treatment. Finally, combining IL‐6 and EGFR blockade successfully controlled pain and tumor growth simultaneously in SWN models (Figure 9).

One of the biggest challenges in SWN research is the lack of clinically relevant models. Schwannomatosis is an umbrella term for conditions associated with the genetic predisposition to multiple schwannomas. SWN has been associated with germline pathogenic variants in the *SMARCB1, LZTR1*, and *NF2* genes, although it is likely that additional predisposition genes exist on chromosome 22q [34]. Genetically engineered mice models (GEMMs) with biallelic inactivation of *Smarcb1* and *Nf2* in the Schwann cell lineage develop tumorlets in the DRG, resembling human schwannoma tumorlets [35]. However, the inconsistent timing of tumor formation and low tumor development rates in GEMMs hinder reproducible, robust drug testing. In the era of personalized medicine, PDX models are of utmost importance. However, as a non‐malignant tumor syndrome, SWN has no reported PDX models. A previous study isolated Schwannoma cells from patients and showed that they secrete inflammatory cytokines and affect gene expression associated with pain in vitro [36]. As a step further, we established two orthotopic PDX models: one mimicking peripheral nerve schwannomas and the other spinal schwannomas. Our models reproduce the pain phenotypes observed in patients with SWN. Additionally, we developed a DRG imaging window model that allows longitudinal, intravital imaging of the DRG microenvironment. These tools address a major bottleneck in developing therapeutics for Schwannomatosis, providing the research and clinical community with robust, biologically diverse models to explore new therapeutic targets for this devastating disease. Future optimization of our PDX models includes: i) breeding the *SMARCB1^−/−^* and *LZTR1^−/−^* GEMM into immunodeficient mouse strains with stromal cells, and innate and adaptive immune cells all harboring the SWN gene mutations, and implanting our patient‐derived SWN cells into the immunodeficient *SMARCB1^−/−^* and *LZTR1^−/−^* GEMM. This could define additional avenues of host contribution to tumor progression and pain response; ii) while nude mice lack functional T cells, they retain innate immune components, including macrophages, although their activity may be somewhat altered compared to immunocompetent mice. However, macrophage function is relatively preserved in nude mice compared to other immunodeficient strains like NOD or NSG mice [37, 38], which have more severe macrophage defects. In future studies, we aim to optimize our PDX model using humanized mice to more accurately evaluate immune contributions to tumor growth and pain, and iii) expanding the SWN cell bank to incorporate lines from more patients, of both sexes, with distinct genetic mutations and pain phenotypes. This would enhance the model's ability to represent disease heterogeneity and enable a deeper understanding of genotype‐pain correlations. However, as SWN is a rare disease and only 2%–5% of patients surgically remove their schwannomas, collaborative efforts across NF clinics will be critical to achieving this goal.

Using the PDX model combined with intravital DRG imaging, we discovered that schwannomas growing distally on the peripheral nerve induced an influx of inflammatory macrophages into the DRG. In this study, we visualized the vasculature and macrophages in the DRG microenvironment, and quantitative analyses of macrophage density yielded robust and reproducible readouts that were independently confirmed by flow cytometry. Dynamic visualization is a powerful advantage of intravital imaging. However, capturing macrophage extravasation events in the DRG presents significant technical challenges. First macrophage extravasation is an asynchronous and relatively rare event, making it difficult to consistently capture video sequences showing macrophage extravasation from blood vessels followed by infiltration into the DRG. Second, DRG intravital imaging requires prolonged anesthesia and strict immobilization, which limit imaging duration. Future optimization of this imaging platform–including enhancing imaging stability and advanced motion correction–will enable high‐quality time‐lapse recordings of inflammatory cell trafficking and allow real‐time visualization of macrophage extravasation and infiltration.

The macrophage DRG infiltration finding prompted us to ask two questions: i) how do distant peripheral nerve tumors cause macrophage infiltration in the DRG? and ii) how do macrophages induce pain in SWN models? Using the orthotopic PDX and DRG imaging models, we addressed these questions and uncovered cellular and molecular crosstalk among schwannoma, neurons, and macrophages in pain generation. We demonstrated that schwannomas secrete HMGB1, which directly induces CCL2 expression in DRG neurons, thereby recruiting macrophages to the DRG and initiating pain. A limitation of our study is that DRG macrophage infiltration was evaluated only in the sciatic nerve model. In the spinal model, the presence of tumor mass within the spinal canal made it challenging to reliably dissect the DRG without contamination from adjacent spinal cord or spinal tumor tissue, preventing accurate flow cytometric analysis. Notably, spinal schwannoma tumors secrete HMGB1 at levels comparable to those observed in sciatic nerve tumors (Figure 5B), suggesting that activation of the tumor (HMGB1)‐neuron (CCL2) signaling axis is not restricted to peripheral tumor location. Nonetheless, direct assessment of DRG immune infiltration in the spinal model was technically challenging and will require future investigation using alternative experimental approaches.

Our findings suggest that CCL2 and HMGB1 are potential therapeutic targets for SWN. HMGB1, a member of the Damage‐Associated Molecular Pattern (DAMP) family, initiates and augments inflammatory response by recruiting inflammatory cells and producing inflammatory cytokines [39, 40, 41, 42]. In our model, **HMGB1** knockdown in schwannoma cells is associated with reduced expression of macrophage‐attracting chemokines in DRG sensory neurons. To determine whether HMGB1 directly regulates neuronal **CCL2** expression, future studies using **HMGB1 receptor knockout mice** are warranted. As **TLR4 knockout mice**, a key HMGB1 receptor, are currently available in an immunocompetent **C57BL/6** background, crossbreeding into an immunodeficient background will be required for use in the patient‐derived SWN model. The observed reduction in pain behavior may also reflect local anti‐inflammatory effects of HMGB1 knockdown on the peripheral nerve. Several strategies targeting HMGB1 and its receptors have shown beneficial effects in preclinical models of inflammatory diseases [43].

CCL2, also known as monocyte chemoattractant protein‐1 (MCP‐1), strongly recruits monocytes and is upregulated in sensory neurons in response to peripheral nerve injury [44, 45, 46, 47]. It can directly enhance pain sensitivity or recruit macrophages to the DRG, causing inflammatory pain [21, 48, 49]. CCL2 also promotes tumor progression and metastasis by stimulating tumor cell proliferation and migration, or indirectly through macrophage‐mediated actions [50, 51, 52, 53]. The dual role of CCL2 in pain and tumor progression explains why anti‐CCL2 treatment reduces both pain and tumor growth, whereas HMGB1 blockade reduces pain only in SWN models. Various agents targeting either CCL2, such as neutralizing antibodies, or its receptor CCR2, such as small‐molecule antagonists, are currently being developed and evaluated for inflammatory diseases and cancers [54]. In schwannomas, tumor‐associated macrophages have been implicated in the pathobiology of *NF2*‐related vestibular schwannomas [55]. Together with our findings demonstrating that anti‐CCL2 treatment reduces DRG inflammation, pain phenotypes, and tumor growth, these observations support a strong translational rationale for targeting the CCL2/CCR2 axis in patients with SWN and sporadic spinal schwannomas. Clinically, CCL2/CCR2 inhibition could be explored either i) as an adjunct therapy to surgery to attenuate macrophage‐driven inflammation and potentially slow tumor progression, and/or ii) as a localized, pain‐focused approach (e.g., intrathecal delivery) to selectively modulate inflammatory signaling near sensory ganglia. Notably, intrathecal anti‐CCL2 has been shown to prevent and partially reverse mechanical hypersensitivity in a DRG‐associated neuropathic pain model [56]. Collectively, these data support CCL2/CCR2 blockade as a promising therapeutic strategy that simultaneously targets tumor‐associated neuroinflammation and pain, warranting further validation in patient‐derived tissues and clinical studies.

Our long‐term goal is to develop therapies that control tumor growth and alleviate pain for SWN patients. We found that SWN‐primed macrophages secret elevated levels of proinflammatory cytokines IL‐6, IL‐1β, and TNFα. The molecular mechanisms by which Schwannoma tumor cells induce these inflammatory cytokine productions in DRG macrophages remain to be elucidated. We focused on IL‐6 blockade because i) it is the most induced cytokine secreted by SWN‐primed macrophages, ii) it is a well‐established mediator of inflammatory diseases and pain [16, 26, 57, 58], and iii) anti‐IL6 (siltuximab) and anti‐IL‐6R antibodies (tocilizumab and sarilumab) are FDA‐approved for pain management in rheumatoid arthritis [29, 30]. In the **painful SCH‐2 model**, which exhibits higher macrophage infiltration, **anti‐CCL2 treatment reduced macrophage numbers in the DRG by approximately 50%** (Figure 4B), resulting in a corresponding reduction in pain behaviors. This partial reduction in macrophage infiltration observed following CCL2 blockade likely reflects functional redundancy within the chemokine network governing macrophage recruitment. In addition to CCL2, several chemokines—including CCL7 (MCP‐3) and CCL12 (MCP‐5)–are known to bind to the CCR2 receptor and can mediate macrophage recruitment, particularly under inflammatory conditions [59, 60]. Thus, residual macrophage infiltration following anti‐CCL2 treatment may be mediated by parallel signaling through these alternative chemokines. These findings suggest that more effective suppression of tumor‐induced neuroinflammation may require combinatorial targeting of multiple chemokines. Notably, **IL‐6 neutralization significantly reduced pain in both models**, suggesting that **IL‐6** plays a broader role in tumor‐induced pain independent of baseline macrophage levels. These results indicate that while CCL2‐driven **macrophage recruitment** contributes to pain in the SCH‐2 model, **IL‐6 signaling** represents the effector mechanism driving pain across different tumors. Intrathecal delivery of analgesics is an established practice for patients with refractory cancer pain, allowing direct administration of agents such as morphine or ziconotide into the cerebrospinal fluid to achieve potent pain control at substantially lower systemic doses. This approach can provide meaningful analgesia when conventional systemic therapies fail, while limiting systemic toxicities. In our mouse PDX model, however, testing repeated intrathecal injection–particularly in the spine model—is technically challenging. Therefore, future clinical studies will be required to evaluate whether targeting IL‐6 signaling via intrathecal or localized delivery strategies can provide effective and durable pain control in patients with SWN. Importantly, based on our preclinical findings, a clinical trial is currently evaluating siltuximab in patients with SWN (NCT05684692), and future studies investigating IL‐1, TNF, and IFN blockade for SWN‐induced pain may offer additional therapeutic options for clinical management.

Our finding that blocking inflammatory IL‐6 signaling does not affect tumor growth suggests that tumorigenesis and pain are mediated through independent mechanisms. We further identified EGF signaling as a driver of SWN tumor progression. The EGFR/ErbB family of receptor tyrosine kinases, which includes EGFR/Her1, Her2 (ErbB2), Her3 (ErbB3), and Her4 (ErbB4) [61], contributes to the progression of many cancers [31, 32]. Targetable ErbB2 mutations have been identified in SWN [62], but the role of ErbB/EGFR in SWN progression remains unclear. In NF2‐related vestibular schwannomas, Lapatinib, an HER‐1/2 inhibitor, reduced tumor size in 4 of 17 patients and improved hearing in 4 of 13 patients [63]. We show that combined IL‐6 and EGFR blockade can reduce pain and inhibit tumor growth, suggesting the need for further clinical studies on this combination strategy.

In summary, using novel PDX and DRG imaging models, we uncovered the cellular and molecular crosstalk between schwannoma (HMGB1)–neuron (CCL2)–macrophage (IL‐6) in driving pain response, and identified the EGF pathway as a critical driver of SWN tumor progression. These findings pave the way for targeted therapies that simultaneously control tumor growth and alleviate pain in SWN patients.

## 4 Experimental Section/Methods

### 4.1 Reagents

Dulbecco's Modified Eagle's Medium and Ham's F‐12 50/50 Mix was obtained from Corning (Manassas, VA). Leibovitz's L‐15 Medium (L15), Neurobasal TM‐A Medium (Neurobasal A), and 2% B‐27 Supplement (50X) were obtained from Thermo Fisher Scientific (Cambridge, MA). Collagen type I, High Concentration, was obtained from Corning (Bedford, MA). Poly‐L‐lysine solution was obtained from Sigma–Aldrich (Natick, MA).

### 4.2 Patient‐derived SWN Cell Lines

Schwannomatosis patient samples were obtained from the Departments of Surgery and Pathology at Massachusetts General Hospital (MGH) from December 2016 through July 2022 under an approved IRB protocol. Patients were diagnosed with Schwannomatosis according to established clinical and genetic criteria [2]. All patients provided signed informed consent for the collection of excess tumor samples and molecular analysis. The indication for surgery in 5/9 patients was pain related to the tumor in question (Table S1). There was no correlation between tumor size and pain (Figure S1A). In a laminar flow hood, the tissue was finely minced and centrifuged for 5 min at 1000 rpm. To dissociate the cells, the tissue was digested with 1 mg/mL collagenase for 3 h at 37°C, then filtered and cultured in SCM medium. Three days later, cells were immortalized using pLenti SV40 (Abm, Richmond, BC) according to the manufacturer's instructions [64]. To confirm their Schwann cell origin, 4% paraformaldehyde (PFA)‐fixed cells were blocked and incubated with anti‐S100 and anti‐Sox10 (1:10, both from Biocare Medical, Pacheco, CA) overnight at 4°C. Alexa 647‐conjugated secondary antibodies were used for signal detection using confocal microscopy. The cells used in the study are free of contamination.

### 4.3 Orthotopic Patient‐derived Xenograft SWN Mouse Models

All animal procedures were performed following the Public Health Service Policy on Humane Care of Laboratory Animals and approved by the Institutional Animal Care and Use Committee of the MGH. Patient‐derived SWN cells were implanted in 8‐12‐weeks‐old immunodeficient nude mice (NU/NU). All nude mice were bred and maintained in the MGH Cox‐7 animal facility. We used an equal number of male and female mice (1:1 ratio) in our experiments, as SWN affects both sexes. All experimental groups were age‐ and sex‐matched to minimize biological variability.

### 4.4 Sciatic Nerve Model

To mimic schwannomas grown on the peripheral nerve, 3 µL of tumor cell suspension (5 × 10^4^ cells/mice) was injected slowly under the sciatic nerve sheath using a Hamilton syringe to prevent leakage [65, 66].

### 4.5 Spine Model

To mimic schwannomas grown in the spine, SWN cells (1 × 10^4^ cells/mice, in 2 µL) were injected intrathecally between T13 and L1. To confirm the formation of tumors in the correct anatomic location and measure tumor growth, we performed 3D ultrasound (US) on the sciatic nerve tumor and MR imaging of the spinal tumor as previously described [65, 67].

### 4.6 Treatment Protocols

In the sciatic nerve model, treatment begins when the tumor reaches 3 mm in diameter. In the spine model, treatment starts when the blood Gaussia luciferase reporter gene (Gluc) concentration reaches 1 × 10^4^ RLU. Treatments included:

*Anti‐CCL2* (2 mg/kg, BioXCell, Cat.#BE0457) or polyclonal Armenian hamster IgG (isotype control, BioXCell, Cat.#BE0091): administered *i.p*. every 3 days.

*Anti‐IL‐6* (5 mg/kg, BioXCell, Cat.#BE0046) or polyclonal rat IgG (BioXCell, Cat.#BE0094): administered *i.p*. every 3 days.

*Dacomitinib* (20 mg/kg, LC Laboratories) or saline (control): administered by oral gavage 5 days/week.

### 4.7 Measurement of Tumor Growth

Tumor cell lines were infected with a lentivirus encoding secreted Gaussia luciferase (Gluc), and plasma Gluc was measured as previously described [68]. Briefly, 13 µL of whole blood was collected from a slight nick on the tail veins and mixed with 5 µL of 50 mm EDTA immediately to avoid clotting. The blood sample was transferred to a 96‐well plate, and Gluc activity was measured using a plate luminometer (GloMax 96 Microplate Luminometer, Promega). The luminometer was set to automatically inject 100 µL of 100 mm coelenterazine (CTZ, Nanolight) in PBS, and photon counts were acquired for 10 s.

### 4.8 Pain Behavior Test and Motor Function Test

Von Frey filament threshold testing: For the mechanical withdrawal threshold test, mice were individually placed in a standard plexiglass enclosure with a mesh grid floor. After 20–30 min of acclimation, mechanical sensitivity was measured using a graded series of von Frey filaments (DanMic Global) applied to the plantar surface of each hind paw using an ascending series. Each filament was tested five times in increasing order, starting with the filament producing the lowest force (0.008 g). Brisk withdrawal of the hind paw, escaping or biting upon the stimuli was defined as a positive withdrawal response, and at least three positive responses out of five applications were defined as the withdrawal threshold, and 2 g was defined as the final threshold if mice exhibited negative responses to all stimuli applications [69, 70, 71]. All testing took place during the light cycle, between 09:00 h and 16:00 h. Although data were collected on both hind paws, only data from the hind paw ipsilateral to the tumor implantation are presented, as no effects of sex, drug, or genotype on the contralateral paw were observed in any experiment.

### 4.9 Rotarod Test

Motor function in the mice was assessed using an automated Rotarod (Columbus Instruments, Columbus, OH). The Rotarod test was performed in animals bearing size‐matched tumors following a previously published protocol [72].

### 4.10 Adoptive Transfer of Bone Marrow‐derived Macrophages

Adoptive transfer experiments were performed using age‐ and sex‐matched donors and recipients to ensure consistency across experimental conditions. We harvested bone marrow cells from *Ccr2^−/−^/*C57BL/6 and *Ccr2^RFP/RFP^/*C57BL/6 mice, and cultured them with M‐CSF (20 ng/mL) for seven days to proliferate and differentiate into macrophages following a previously published protocol [73]. Nude mice (8 weeks of age) were sub‐lethally whole‐body irradiated with a single dose of 6 Gy. Twenty‐four hours later, 1 × 10^6^/in 100 µL donor bone marrow‐derived macrophages were injected retro‐orbitally into the irradiated recipients [66]. Peripheral blood was collected 3 weeks post‐injection and analyzed for RFP^+^ and CD45.2^+^ cells by flow cytometry (CD45, 30F11, BioLegend).

### 4.11 Implantation of DRG Window and Intravital Multiphoton Laser Scanning Microscopy

Mice were anesthetized and placed in a prone position on a temperature‐controlled surgical stage. A small dorsal incision was made at the lumbar (L3‐L4) level, and underlying paraspinal muscles and connective tissues were carefully dissected and retracted under a dissecting microscope to expose the vertebral column. Muscles and ligaments attached to the target vertebrae were gently detached using fine surgical scissors. To provide optical access to the DRG, the articular processes surrounding the DRG were trimmed using a microdrill to a level flush with the DRG surface, while preserving the integrity of the dura and adjacent nerve roots. Hemostasis was carefully maintained throughout the procedure. A custom‐designed, 3D‐printed DRG imaging window incorporating a 3‐mm‐diameter glass coverslip was positioned directly over the exposed DRG and secured to the surrounding vertebral bone with sutures and dental cement, providing stable, optimal access while minimizing motion artifacts. The exposed tissue was continuously irrigated with sterile saline. Following window implantation, animals were allowed to stabilize under anesthesia prior to imaging. Intravital multiphoton laser scanning microscopy was then performed as previously described [65]. Mice were deeply anesthetized with an intraperitoneal injection of 100 mg/kg ketamine and 15 mg/kg xylazine. We performed retro‐orbital injection of FITC‐Dextran (10 mg/mL, FisherThermo, Cat# FD200S) as a vascular contrast agent. Mice were put under the two‐photon microscope to image macrophages.

### 4.12 HMGB1 Knockout using CRISPR‐Cas9

The HMGB1 CRISPR/Cas knockout (KO) plasmid (Santa Cruz Biotechnology, sc‐400735) consisted of a pool of three plasmids, each encoding the Cas9 nuclease, and HMGB‐1‐specific 20‐nt guide RNA (gRNA) for maximum KO efficiency. After plasmid transfection, single‐cell clones were picked following antibiotic selection and screened for HMGB1 knockdown using ELISA (R&D Systems). To avoid clonal variation, 3 clones with the lowest HMGB1 expression were pooled for use in animal experiments.

### 4.13 Organotypic DRG Ex Vivo Culture

DRGs were surgically dissected from 1–2‐day‐old postnatal nude mice. The DRGs, collected from L1‐L6 lumbar vertebrae, were transferred to L15 medium. They were then cultured in a slide chamber coated with poly‐L‐lysine and collagen.

### 4.14 Flow Cytometry Analysis of DRG Immune Cells

Lumbar DRGs (L3‐L6), ipsilateral to the sciatic nerve tumors or bilaterally in the spine model, were harvested from mice following transcardial perfusion with cold PBS to remove circulating immune cells. DRGs were enzymatically dissociated into single‐cell suspensions. Due to the limited cell yield, DRGs from 3 mice per group were pooled to ensure sufficient event analysis. Cells were stained with viability dye and fluorophore‐conjugated antibodies against CD45 (pan‐leukocyte, BioLegend, Cat.#103140), CD11b (myeloid cell, BioLegend, Cat.#101243), F4/80 (macrophages, BioLegend, Cat.#111604), CD206 (M2 marker, BioLegend, Cat.#141706), and MHCII (M1 marker, BioLegend, Cat.# 107630). All data were collected on a BD LSR Fortessa or FACSAria IIu (BD Bioscience) instrument. Flow cytometry data were analyzed using FlowJo software. Immune populations were identified through sequential gating on singlets, live cells, and CD45^+^ immune cells. Macrophages were defined as CD45^+^CD11b^+^F4/80^+^; M1‐like and M2‐like subsets were identified by MHCII and CD206 expression, respectively. Given the low cell numbers, data are presented as percentages of CD45^+^ cells rather than absolute counts. Gating strategies are provided in Supplemental Figure S2D–E.

### 4.15 Gene Expression Analysis

Gene expression was analyzed at the RNA level by RNASeq, cDNA array, and quantitative RT‐PCR (qPCR); and at the protein level by western blot and multiplex ELISA following routine procedure in the Xu lab [66, 74, 75, 76, 77, 78]. Primers used for qPCR are: *muCcl2*: forward‐GTC TGT GCT GAC CCC AAG AAG, and reverse‐TGG TTC CGA TCC AGG TTT TTA; *muGapdh*: forward‐AGG TCG GTG TGA ACG GAT TTG, and reverse–TGT AGA CCA TGT AGT TGA GGT CA. For western blot, membranes were blotted with antibodies against total (1:500) and phospho‐ErbB2 (1:1000); total (1:500) and phospho‐ErbB3 (1:500); total (1:500) and phospho‐ErbB4 (1:500); total (1:1000) and phospho‐Akt (1:200); total and phospho‐S6 (1:1000 for both). Antibodies were obtained from Cell Signaling (Danvers, MA). Membranes were blotted with antibody against beta‐actin for equal loading control (1:5000, Sigma).

### 4.16 MTT Assay

Five thousand cells were seeded into 38 mm^2^ wells of flat‐bottomed 96‐well plates in triplicate and allowed to adhere overnight. 3‐(4,5‐Dimethylthiazol‐2‐yl)‐2,5‐diphenyltetrazolium bromide (MTT, 5 mg/mL, Sigma Chemical) was prepared in PBS. The number of metabolically active cells was determined by MTT assay [79].

### 4.17 Histological staining

DRG slices were fixed with 4% PFA and 20% sucrose, blocked in 5% BSA for 1 h, and then incubated against anti‐CGRP (1:5000), anti‐TRPV1 (1:800), and anti‐IB4 (1:500), all antibodies were ordered from ThermoFisher. To evaluate tumor cell proliferation and apoptosis, slides of tumor tissues were stained with proliferating cell nuclear antigen (PCNA, 1:1000, Abcam) and TUNEL (ApopTag, EMD Millipore). Archived paraffin‐embedded Schwannomatosis patient samples were stained with antibodies against CD163 (Dako), IL‐6 (1:100, Abcam), and phosphor‐Stat3 (1:10, Cell Signaling Technology). Four specimens of normal peripheral nerve were obtained postmortem and used as controls. Appropriate positive and negative controls were used for all stains [78].

### 4.18 Image Quantification

For the macrophage, PCNA, and TUNEL staining, positively stained cells are manually counted. The positively stained area in all other histological stains was evaluated using digital quantitative image analysis with the open‐source software ImageJ. Positive staining in 20 random fields/slides was quantified using built‐in automated functions based on fluorescent pixel intensity after establishing a threshold to exclude background staining. Individual staining was quantified as the area fraction of the tumor region of interest and reported as a percentage [66].

### 4.19 Statistical Analyses

Tumor growth was analyzed using two‐way repeated‐measures ANOVA to assess treatment effects across groups. Pain behavior tests (von Frey filament test, spontaneous lifting and licking test) were analyzed using unpaired two‐tailed t‐tests, with comparisons made between groups at each time point. For gene expression, flow cytometry, in vitro viability assay, and image quantification comparison, significant differences between the two groups were determined using an unpaired two‐tailed Student's t‐test. All calculations were done using GraphPad Prism Software 6.0 and Microsoft Excel Software 2010.

## Author Contributions

L.X. designed the research and supervised the research; Z.Y., L.W., and Y.Z. established patient‐derived cell lines; Z.Y., Y.S., and S.L., performed mouse model studies; Z.Y., J.R., G.X., and J.W.C. performed imaging studies; S.S. and W.H. analyzed RNASeq data; G.Y.L. design and 3‐D printed the DRG window and created the Graphic Abstract; Y.Z. and G.B.F. established the DRG ex vivo culture model; L.W., Y.S., and A.S.R. performed patient sample histological analysis; L.X., Z.Y., L.W., Y.Z., Y.S., J.W.C., S.S., W.H., A.M., and A.S.R. analyzed data; L.X., S.R.P., and J.M. wrote the paper.

## Funding

This study was supported by the NIH R01‐NS126187 (to L.X. and J.M.) and R01‐DC020724 (to L.X.), Department of Defense New Investigator Award (W81XWH‐16‐1‐0219, to L.X.), Investigator‐Initiated Research Award (W81XWH‐20‐1‐0222, to L.X.), Clinical Trial Award (W81XWH2210439, to S.R.P. and L.X.), Children's Tumor Foundation Drug Discovery Initiative (to L.X.), Children's Tumor Foundation Clinical Research Award (to L.X. and S.R.P.), American Cancer Society Mission Boost Award (to L.X. doi.org/10.53354/ACS.MBGII‐24‐1255260‐01‐MBG.pc.gr.193735), and R01‐NS103998 (to J.W.C.), NIH‐NINDS: R01 grants: (NS126187, DC020724).

## Conflicts of Interest

The authors declare no conflicts of interest.

## Supporting information




**Supporting File**: advs74584‐sup‐0001‐SuppMat.pdf.

## Data Availability

The data that support the findings of this study are available from the corresponding author upon reasonable request.

## References

[1] D. G. Evans , N. L. Bowers , S. Tobi , et al., “Schwannomatosis: A Genetic and Epidemiological Study,” Journal of Neurology, Neurosurgery & Psychiatry 89 (2018): 1215–1219, 10.1136/jnnp-2018-318538. 29909380

[2] S. R. Plotkin , L. Messiaen , E. Legius , et al., “Updated Diagnostic Criteria and Nomenclature for Neurofibromatosis Type 2 and Schwannomatosis: An International Consensus Recommendation,” Genetics in Medicine 24 (2022): 1967–1977, 10.1016/j.gim.2022.05.007. 35674741

[3] T. J. Hulsebos , A. S. Plomp , R. A. Wolterman , E. C. Robanus‐Maandag , F. Baas , and P. Wesseling , “Germline Mutation of INI1/SMARCB1 in Familial Schwannomatosis,” The American Journal of Human Genetics 80 (2007): 805–810, 10.1086/513207. 17357086 PMC1852715

[4] A. Piotrowski , J. Xie , Y. F. Liu , et al., “Germline Loss‐of‐function Mutations in LZTR1 Predispose to an Inherited Disorder of Multiple Schwannomas,” Nature Genetics 46 (2014): 182–187, 10.1038/ng.2855. 24362817 PMC4352302

[5] M. J. Smith , B. Isidor , C. Beetz , et al., “Mutations in LZTR1 Add to the Complex Heterogeneity of Schwannomatosis,” Neurology 84 (2015): 141–147, 10.1212/WNL.0000000000001129. 25480913 PMC4336087

[6] M. J. Smith , A. J. Wallace , N. L. Bowers , et al., “Frequency of SMARCB1 Mutations in Familial and Sporadic Schwannomatosis,” Neurogenetics 13 (2012): 141–145, 10.1007/s10048-012-0319-8. 22434358

[7] M. MacCollin , E. A. Chiocca , D. G. Evans , et al., “Diagnostic Criteria for Schwannomatosis,” Neurology 64 (2005): 1838–1845, 10.1212/01.WNL.0000163982.78900.AD. 15955931

[8] A. Gonzalvo , A. Fowler , R. J. Cook , et al., “Schwannomatosis, sporadic schwannomatosis, and familial schwannomatosis: A surgical series With long‐term follow‐up,” Journal of Neurosurgery 114 (2011): 756–762, 10.3171/2010.8.JNS091900. 20932094

[9] V. L. Merker , S. Esparza , M. J. Smith , A. Stemmer‐Rachamimov , and S. R. Plotkin , “Clinical Features of Schwannomatosis: A Retrospective Analysis of 87 Patients,” The Oncologist 17 (2012): 1317–1322, 10.1634/theoncologist.2012-0162. 22927469 PMC3481897

[10] C. Lu‐Emerson and S. R. Plotkin , “The Neurofibromatoses. Part 2: NF2 and Schwannomatosis,” Reviews in Neurological Diseases 6 (2009): E81–86. 19898272

[11] J. O. Blakeley and S. R. Plotkin , “Therapeutic Advances for the Tumors Associated with Neurofibromatosis Type 1, Type 2, and Schwannomatosis,” Neuro‐Oncology 18 (2016): 624–638, 10.1093/neuonc/nov200. 26851632 PMC4827037

[12] J. Blakeley , K. C. Schreck , D. G. Evans , et al., “Clinical Response to bevacizumab in Schwannomatosis,” Neurology 83 (2014): 1986–1987, 10.1212/WNL.0000000000000997. 25339217 PMC4248457

[13] S. R. Plotkin , J. O. Blakeley , D. G. Evans , et al., “Update From the 2011 International Schwannomatosis Workshop: From Genetics to Diagnostic Criteria,” American Journal of Medical Genetics Part A 161 (2013): 405–416, 10.1002/ajmg.a.35760. PMC4020435 23401320

[14] N. T. Fiore , S. R. Debs , J. P. Hayes , S. S. Duffy , and G. Moalem‐Taylor , “Pain‐resolving Immune Mechanisms in Neuropathic Pain,” Nature reviews Neurology 19 (2023): 199–220. 36859719 10.1038/s41582-023-00777-3

[15] D. B. Reichling , P. G. Green , and J. D. Levine , “The Fundamental Unit of Pain Is the Cell,” Pain 154 (2013): S2–S9, 10.1016/j.pain.2013.05.037. 23711480 PMC3858489

[16] O. A. Dina , T. Hucho , J. Yeh , M. Malik‐Hall , D. B. Reichling , and J. D. Levine , “Primary Afferent Second Messenger Cascades Interact With Specific Integrin Subunits in Producing Inflammatory Hyperalgesia,” Pain 115 (2005): 191–203, 10.1016/j.pain.2005.02.028. 15836982

[17] X. Yu , H. Liu , K. A. Hamel , et al., “Dorsal Root Ganglion Macrophages Contribute to both the Initiation and Persistence of Neuropathic Pain,” Nature Communications 11 (2020): 264, 10.1038/s41467-019-13839-2. PMC6959328 31937758

[18] F. C. Richter and I. Udalova , “Macrophage Commonalities Across Tissues and Inflammation,” Nature Reviews Immunology 22 (2022): 2, 10.1038/s41577-021-00659-z. 34795417

[19] K. Biber and E. Boddeke , “Neuronal CC Chemokines: The Distinct Roles of CCL21 and CCL2 in Neuropathic Pain,” Frontiers in Cellular Neuroscience 8 (2014): 210, 10.3389/fncel.2014.00210. 25147499 PMC4124792

[20] R. Xie , Y. Gao , C. Park , et al., “Spinal CCL2 Promotes Central Sensitization, Long‐Term Potentiation, and Inflammatory Pain via CCR2: Further Insights Into Molecular, Synaptic, and Cellular Mechanisms,” Neuroscience Bulletin 34 (2018): 13–21, 10.1007/s12264-017-0106-5. 28265898 PMC5587365

[21] O. Bogen , O. A. Dina , R. W. Gear , and J. D. Levine , “Dependence of Monocyte Chemoattractant Protein 1 Induced Hyperalgesia on the Isolectin B4‐binding Protein versican,” Neuroscience 159 (2009): 780–786, 10.1016/j.neuroscience.2008.12.049. 19167466 PMC2684808

[22] C. L. Hsieh , E. C. Niemi , S. H. Wang , et al., “CCR2 deficiency Impairs Macrophage Infiltration and Improves Cognitive Function After Traumatic Brain Injury,” Journal of Neurotrauma 31 (2014): 1677–1688, 10.1089/neu.2013.3252. 24806994 PMC4545982

[23] J. Cai , H. Yuan , Q. Wang , et al., “HMGB1‐Driven Inflammation and Intimal Hyperplasia After Arterial Injury Involves Cell‐Specific Actions Mediated by TLR4,” Arteriosclerosis, Thrombosis, and Vascular Biology 35 (2015): 2579–2593, 10.1161/ATVBAHA.115.305789. 26515416 PMC4880018

[24] D. P. Kodack , V. Askoxylakis , G. B. Ferraro , et al., “The Brain Microenvironment Mediates Resistance in Luminal Breast Cancer to PI3K Inhibition Through HER3 Activation,” Science Translational Medicine 9 (2017), 10.1126/scitranslmed.aal4682. PMC5917603 28539475

[25] L. Xu , K. Xie , and I. J. Fidler , “Therapy of Human Ovarian Cancer by Transfection with the Murine Interferon β Gene: Role of Macrophage‐Inducible Nitric Oxide Synthase,” Human Gene Therapy 9 (1998): 2699–2708, 10.1089/hum.1998.9.18-2699. 9874268

[26] T. Alonzi , E. Fattori , D. Lazzaro , et al., “Interleukin 6 Is Required for the Development of Collagen‐induced Arthritis,” The Journal of Experimental Medicine 187 (1998): 461–468, 10.1084/jem.187.4.461. 9463396 PMC2212160

[27] D. Fang , L. Kong , J. Cai , et al., “Interleukin‐6‐mediated functional upregulation of TRPV1 receptors in dorsal root ganglion neurons Through the activation of JAK/PI3K signaling pathway,” Pain 156 (2015): 1124–1144, 10.1097/j.pain.0000000000000158. 25775359

[28] O. A. Dina , P. G. Green , and J. D. Levine , “Role of Interleukin‐6 in Chronic Muscle Hyperalgesic Priming,” Neuroscience 152 (2008): 521–525, 10.1016/j.neuroscience.2008.01.006. 18280048 PMC2336107

[29] S. A. Jones , J. Scheller , and S. Rose‐John , “Therapeutic Strategies for the Clinical Blockade of IL‐6/gp130 Signaling,” Journal of Clinical Investigation 121 (2011): 3375–3383, 10.1172/JCI57158. 21881215 PMC3163962

[30] E. H. Choy , F. De Benedetti , T. Takeuchi , M. Hashizume , M. R. John , and T. Kishimoto , “Translating IL‐6 Biology into Effective Treatments,” Nature Reviews Rheumatology 16 (2020): 335–345, 10.1038/s41584-020-0419-z. 32327746 PMC7178926

[31] N. Normanno , A. De Luca , C. Bianco , et al., “Epidermal Growth Factor Receptor (EGFR) Signaling in Cancer,” Gene 366 (2006): 2–16, 10.1016/j.gene.2005.10.018. 16377102

[32] A. Passaro , P. A. Janne , T. Mok , and S. Peters , “Overcoming Therapy Resistance in EGFR‐mutant Lung Cancer,” Nature Cancer 2 (2021): 377–391, 10.1038/s43018-021-00195-8. 35122001

[33] R. Roskoski , “Small Molecule Inhibitors Targeting the EGFR/ErbB family of Protein‐tyrosine Kinases in human Cancers,” Pharmacological Research 139 (2019): 395–411, 10.1016/j.phrs.2018.11.014. 30500458

[34] S. R. Plotkin and A. Wick , “Neurofibromatosis and Schwannomatosis,” Seminars Neurology 38 (2018): 73–85. 10.1055/s-0038-1627471 29548054

[35] J. Vitte , F. Gao , G. Coppola , A. R. Judkins , and M. Giovannini , “Timing of Smarcb1 and Nf2 Inactivation Determines Schwannoma versus Rhabdoid Tumor Development,” Nature Communications 8 (2017): 300, 10.1038/s41467-017-00346-5. PMC5563506 28824165

[36] K. L. Ostrow , K. J. Donaldson , M. J. Caterina , A. Belzberg , and A. Hoke , “The Secretomes of Painful versus Nonpainful Human Schwannomatosis Tumor Cells Differentially Influence Sensory Neuron Gene Expression and Sensitivity,” Scientific Reports 9 (2019): 13098. 31511601 10.1038/s41598-019-49705-w PMC6739480

[37] D. V. Serreze , J. W. Gaedeke , and E. H. Leiter , “Hematopoietic Stem‐cell Defects Underlying Abnormal Macrophage Development and Maturation in NOD/Lt Mice: Defective Regulation of Cytokine Receptors and Protein Kinase C,” Proceedings of the National Academy of Sciences 90 (1993): 9625–9629, 10.1073/pnas.90.20.9625. PMC47622 8415751

[38] L. D. Shultz , P. A. Schweitzer , S. W. Christianson , et al., “Multiple Defects in Innate and Adaptive Immunologic Function in NOD/LtSz‐scid Mice,” The Journal of Immunology 154 (1995): 180–191, 10.4049/jimmunol.154.1.180. 7995938

[39] E. Venereau , M. Casalgrandi , M. Schiraldi , et al., “Mutually Exclusive Redox Forms of HMGB1 Promote Cell Recruitment or Proinflammatory Cytokine Release,” Journal of Experimental Medicine 209 (2012): 1519–1528, 10.1084/jem.20120189. 22869893 PMC3428943

[40] E. Venereau , M. Schiraldi , M. Uguccioni , and M. E. Bianchi , “HMGB1 and Leukocyte Migration During Trauma and Sterile Inflammation,” Molecular Immunology 55 (2013): 76–82, 10.1016/j.molimm.2012.10.037. 23207101

[41] H. Wang , O. Bloom , M. Zhang , et al., “HMG‐1 as a Late Mediator of Endotoxin Lethality in Mice,” Science 285 (1999): 248–251, 10.1126/science.285.5425.248. 10398600

[42] Y. N. Paudel , M. F. Shaikh , A. Chakraborti , et al., “HMGB1: A Common Biomarker and Potential Target for TBI, Neuroinflammation, Epilepsy, and Cognitive Dysfunction,” Frontiers in Neuroscience 12 (2018): 628, 10.3389/fnins.2018.00628. 30271319 PMC6142787

[43] H. Yang , H. Wang , and U. Andersson , “Targeting Inflammation Driven by HMGB1,” Frontiers in Immunology 11 (2020): 484, 10.3389/fimmu.2020.00484. 32265930 PMC7099994

[44] T. Tanaka , M. Minami , T. Nakagawa , and M. Satoh , “Enhanced Production of Monocyte Chemoattractant Protein‐1 in the Dorsal Root Ganglia in a Rat Model of Neuropathic Pain: Possible Involvement in the Development of Neuropathic Pain,” Neuroscience Research 48 (2004): 463–469, 10.1016/j.neures.2004.01.004. 15041200

[45] F. A. White , J. Sun , S. M. Waters , et al., “Excitatory Monocyte Chemoattractant Protein‐1 Signaling Is Up‐regulated in Sensory Neurons After Chronic Compression of the Dorsal Root Ganglion,” Proceedings of the National Academy of Sciences 102 (2005): 14092–14097, 10.1073/pnas.0503496102. PMC1236537 16174730

[46] S. M. Jeon , K. M. Lee , E. S. Park , Y. H. Jeon , and H. J. Cho , “Monocyte Chemoattractant Protein‐1 Immunoreactivity in Sensory Ganglia and Hindpaw After Adjuvant Injection,” Neuroreport 19 (2008): 183–186, 10.1097/WNR.0b013e3282f3c781. 18185105

[47] G. Lazennec and A. Richmond , “Chemokines and Chemokine Receptors: New Insights into Cancer‐related Inflammation,” Trends in Molecular Medicine 16 (2010): 133–144, 10.1016/j.molmed.2010.01.003. 20163989 PMC2840699

[48] J. H. Sun , B. Yang , D. F. Donnelly , C. Ma , and R. H. LaMotte , “MCP‐1 Enhances Excitability of Nociceptive Neurons in Chronically Compressed Dorsal Root Ganglia,” Journal of Neurophysiology 96 (2006): 2189–2199, 10.1152/jn.00222.2006. 16775210

[49] G. E. White , A. J. Iqbal , and D. R. Greaves , “CC Chemokine Receptors and Chronic Inflammation—Therapeutic Opportunities and Pharmacological Challenges,” Pharmacological Reviews 65 (2013): 47–89, 10.1124/pr.111.005074. 23300131

[50] P. Pathria , T. L. Louis , and J. A. Varner , “Targeting Tumor‐Associated Macrophages in Cancer,” Trends in Immunology 40 (2019): 310–327, 10.1016/j.it.2019.02.003. 30890304

[51] R. D. Loberg , C. Ying , M. Craig , et al., “Targeting CCL2 With Systemic Delivery of Neutralizing Antibodies Induces Prostate Cancer Tumor Regression in Vivo,” Cancer Research 67 (2007): 9417–9424, 10.1158/0008-5472.CAN-07-1286. 17909051

[52] R. D. Loberg , L. L. Day , J. Harwood , et al., “CCL2 is a Potent Regulator of Prostate Cancer Cell Migration and Proliferation,” Neoplasia 8 (2006): 578–586, 10.1593/neo.06280. 16867220 PMC1601934

[53] M. Xu , Y. Wang , R. Xia , Y. Wei , and X. Wei , “Role of the CCL2‐CCR2 signalling axis in cancer: Mechanisms and therapeutic targeting,” Cell Proliferation 54 (2021): 13115, 10.1111/cpr.13115. PMC8488570 34464477

[54] S. Kadomoto , K. Izumi , and A. Mizokami , “Roles of CCL2‐CCR2 Axis in the Tumor Microenvironment,” International Journal of Molecular Sciences 22 (2021): 8530, 10.3390/ijms22168530. 34445235 PMC8395188

[55] C. J. Hannan , D. Lewis , C. O'Leary , et al., “Increased Circulating Chemokines and Macrophage Recruitment in Growing Vestibular Schwannomas,” Neurosurgery 92 (2023): 581–589, 10.1227/neu.0000000000002252. 36729787

[56] A. M. Illias , A. C. Gist , H. Zhang , A. K. Kosturakis , and P. M. Dougherty , “Chemokine CCL2 and Its Receptor CCR2 in the Dorsal Root Ganglion Contribute to Oxaliplatin‐induced Mechanical Hypersensitivity,” Pain 159 (2018): 1308–1316, 10.1097/j.pain.0000000000001212. 29554018 PMC6008166

[57] R. Atreya , J. Mudter , S. Finotto , et al., “Blockade of Interleukin 6 Trans Signaling Suppresses T‐cell Resistance Against Apoptosis in Chronic Intestinal Inflammation: Evidence in crohn Disease and Experimental Colitis in Vivo,” Nature Medicine 6 (2000): 583–588, 10.1038/75068. 10802717

[58] K. M. Andrejko , N. R. Raj , P. K. Kim , M. Cereda , and C. S. Deutschman , “IL‐6 Modulates Sepsis‐induced Decreases in Transcription of Hepatic Organic Anion and Bile Acid Transporters,” Shock (Augusta, Ga) 29 (2008): 490–496, 10.1097/SHK.0b013e318150762b. 17724432 PMC2667679

[59] X. Li , Y. Long , Y. Zhu , J. Gu , P. Zhou , and C. Miao , “Endothelial‐Derived CCL7 Promotes Macrophage Polarization and Aggravates Septic Acute Lung Injury via CCR1‐Mediated STAT1 Succinylation,” Advanced Science 12 (2025): 06209, 10.1002/advs.202506209. PMC12520477 40755420

[60] L. Sanchez‐Martin , A. Estecha , R. Samaniego , S. Sanchez‐Ramon , M. A. Vega , and P. Sanchez‐Mateos , “The Chemokine CXCL12 Regulates Monocyte‐macrophage Differentiation and RUNX3 Expression,” Blood 117 (2011): 88–97, 10.1182/blood-2009-12-258186. 20930067

[61] C. S. Cancer , “Combating Resistance to EGFR Inhibitors,” Nature Reviews Drug Discovery 18 (2018): 18. 10.1038/nrd.2018.232 30591719

[62] M. W. Ronellenfitsch , P. N. Harter , M. Kirchner , et al., “Targetable ERBB2 Mutations Identified in Neurofibroma/Schwannoma Hybrid Nerve Sheath Tumors,” Journal of Clinical Investigation 130 (2020): 2488–2495, 10.1172/JCI130787. 32017710 PMC7190903

[63] M. A. Karajannis , G. Legault , M. Hagiwara , et al., “Phase II Trial of lapatinib in Adult and Pediatric Patients With Neurofibromatosis Type 2 and Progressive Vestibular Schwannomas,” Neuro‐Oncology 14 (2012): 1163–1170, 10.1093/neuonc/nos146. 22844108 PMC3424212

[64] J. Liu , S. Liao , Y. Huang , et al., “PDGF‐D Improves Drug Delivery and Efficacy via Vascular Normalization, but Promotes Lymphatic Metastasis by Activating CXCR4 in Breast Cancer,” Clinical Cancer Research 17 (2011): 3638–3648, 10.1158/1078-0432.CCR-10-2456. 21459800 PMC3107920

[65] X. Gao , Y. Zhao , A. O. Stemmer‐Rachamimov , et al., “Anti‐VEGF Treatment Improves Neurological Function and Augments Radiation Response in NF2 Schwannoma Model,” Proceedings of the National Academy of Sciences 112 (2015): 14676–14681, 10.1073/pnas.1512570112. PMC4664377 26554010

[66] L. Wu , S. Vasilijic , Y. Sun , et al., “Losartan Prevents Tumor‐induced Hearing Loss and Augments Radiation Efficacy in NF2 Schwannoma Rodent Models,” Science Translational Medicine 13 (2021), 10.1126/scitranslmed.abd4816. PMC8409338 34261799

[67] J. Chen , L. D. Landegger , Y. Sun , et al., “A Cerebellopontine Angle Mouse Model for the Investigation of Tumor Biology, Hearing, and Neurological Function in NF2‐related Vestibular Schwannoma,” Nature Protocols 14 (2019): 541–555, 10.1038/s41596-018-0105-7. 30617350 PMC6571021

[68] Y. Zhao , P. Liu , N. Zhang , et al., “Targeting the cMET Pathway Augments Radiation Response Without Adverse Effect on Hearing in NF2 Schwannoma Models,” Proceedings of the National Academy of Sciences 115 (2018): E2077–E2084, 10.1073/pnas.1719966115. PMC5834719 29440379

[69] S. Shen , G. Lim , Z. You , et al., “Gut Microbiota Is Critical for the Induction of Chemotherapy‐induced Pain,” Nature Neuroscience 20 (2017): 1213–1216, 10.1038/nn.4606. 28714953 PMC5575957

[70] S. L. Christensen , R. B. Hansen , M. A. Storm , et al., “Von Frey testing revisited: Provision of an online algorithm for improved accuracy of 50% thresholds,” European Journal of Pain 24 (2020): 783–790, 10.1002/ejp.1528. 31889375

[71] J. R. Deuis , L. S. Dvorakova , and I. Vetter , “Methods Used to Evaluate Pain Behaviors in Rodents,” Frontiers in Molecular Neuroscience 10 (2017): 284, 10.3389/fnmol.2017.00284. 28932184 PMC5592204

[72] N. Zhang , J. Chen , G. B. Ferraro , et al., “Anti‐VEGF Treatment Improves Neurological Function in Tumors of the Nervous System,” Experimental Neurology 299 (2018): 326–333, 10.1016/j.expneurol.2017.09.008. 28911884

[73] J. Weischenfeldt and B. Porse , “Bone Marrow‐Derived Macrophages (BMM): Isolation and Applications,” CSH protocols 2008 (2008): prot5080. 10.1101/pdb.prot5080 21356739

[74] S. Liao , J. Liu , P. Lin , T. Shi , R. K. Jain , and L. Xu , “TGF‐β Blockade Controls Ascites by Preventing Abnormalization of Lymphatic Vessels in Orthotopic Human Ovarian Carcinoma Models,” Clinical Cancer Research 17 (2011): 1415–1424, 10.1158/1078-0432.CCR-10-2429. 21278244 PMC3060297

[75] Y. Zhao , J. Cao , A. Melamed , et al., “Losartan Treatment Enhances Chemotherapy Efficacy and Reduces Ascites in Ovarian Cancer Models by Normalizing the Tumor Stroma,” Proceedings of the National Academy of Sciences 116 (2019): 2210–2219, 10.1073/pnas.1818357116. PMC6369817 30659155

[76] L. Xu , P. S. Pathak , and D. Fukumura , “Hypoxia‐Induced Activation of p38 Mitogen‐Activated Protein Kinase and Phosphatidylinositol 3′‐Kinase Signaling Pathways Contributes to Expression of Interleukin 8 in Human Ovarian Carcinoma Cells,” Clinical Cancer Research 10 (2004): 701–707, 10.1158/1078-0432.CCR-0953-03. 14760093

[77] L. Xu , D. G. Duda , E. di Tomaso , et al., “Direct Evidence that Bevacizumab, an Anti‐VEGF Antibody, Up‐regulates SDF1α, CXCR4, CXCL6, and Neuropilin 1 in Tumors From Patients With Rectal Cancer,” Cancer Research 69 (2009): 7905–7910, 10.1158/0008-5472.CAN-09-2099. 19826039 PMC2859041

[78] L. Xu , D. M. Cochran , R. T. Tong , et al., “Placenta Growth Factor Overexpression Inhibits Tumor Growth, Angiogenesis, and Metastasis by Depleting Vascular Endothelial Growth Factor Homodimers in Orthotopic Mouse Models,” Cancer Research 66 (2006): 3971–3977, 10.1158/0008-5472.CAN-04-3085. 16618713

[79] L. Xu , R. Tong , D. M. Cochran , and R. K. Jain , “Blocking Platelet‐Derived Growth Factor‐D/Platelet‐Derived Growth Factor Receptor β Signaling Inhibits Human Renal Cell Carcinoma Progression in an Orthotopic Mouse Model,” Cancer Research 65 (2005): 5711–5719, 10.1158/0008-5472.CAN-04-4313. 15994946

