# Supplementary material for: Deciphering and Targeting the Schwannoma‐Neuron‐Macrophage Crosstalk for the Treatment of Schwannomatosis and Associated Pain
Source: Adv Sci (Weinh). 2026 Feb 27;13(26):e15597. doi: 10.1002/advs.202515597 (PMC13159156; doi:10.1002/advs.202515597)
Supplement: Supplementary file 1 — Supporting File: advs74584‐sup‐0001‐SuppMat.pdf. [file ADVS-13-e15597-s001.pdf]

Deciphering and targeting the schwannoma-neuron-macrophage crosstalk for the treatment of schwannomatosis and associated pain

Authors

Zhenzhen Yin[1], Limeng Wu[1,a], Yanling Zhang[1,b], Yao Sun[1,c], Grace Y. Lee[2], Simeng Lu[1], Xing Gao[1,d], John W. Chen[3], Sonu Subudhi[1], William Ho[1], Chao Zhu[1,d], Jun Ren[1], Gino B. Ferraro[1], Alona Muzikansky[4], Anat Stemmer-Rachamimov[5], Jianren Mao[6], Scott R. Plotkin[7], and Lei Xu[1,*]

Supplementary Figures

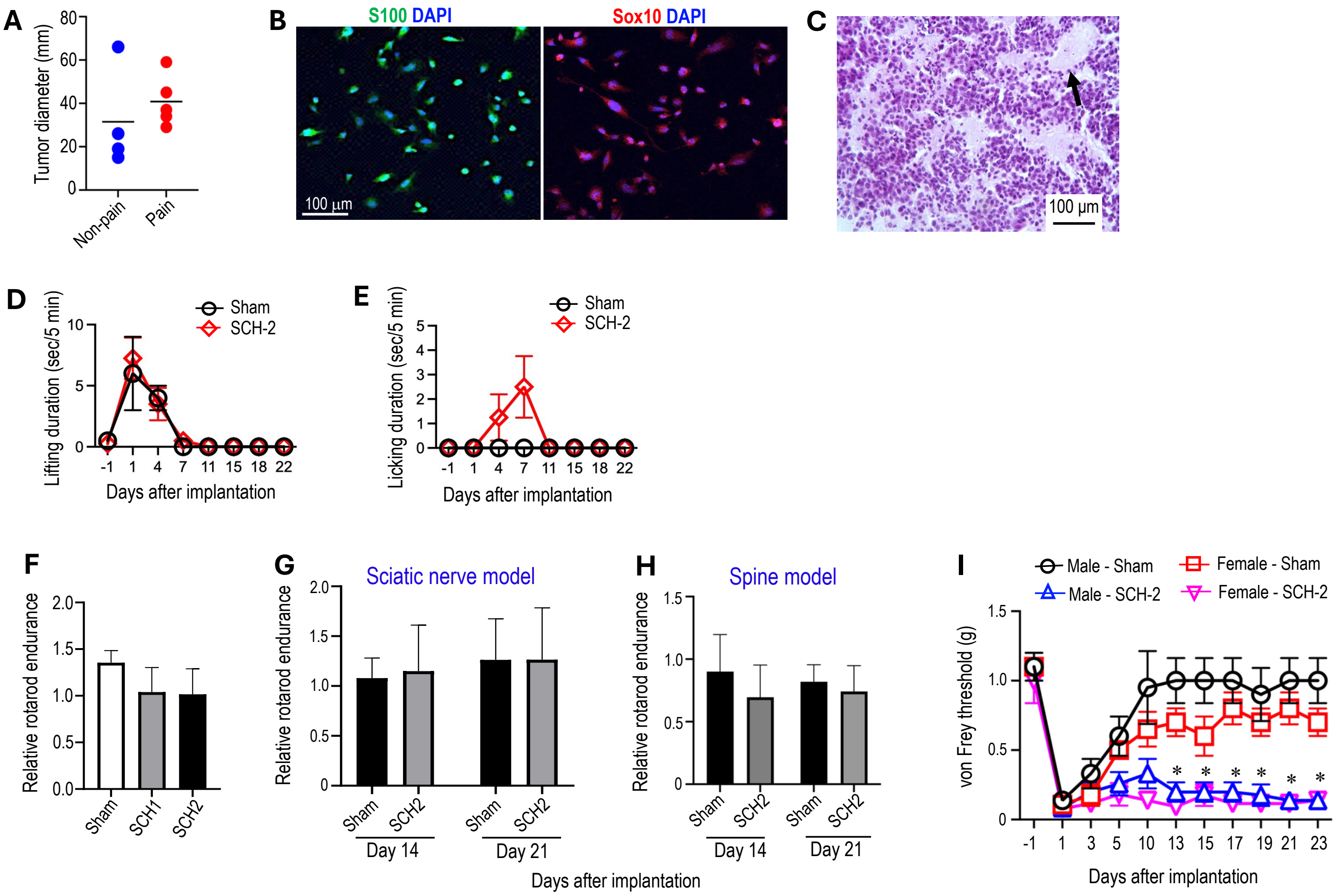

Figure S1. Characterization of patient-derived PDX SWN models. (A) Comparison of tumor diameter at the time of surgery from painful and non-painful SWN patients. See Table S1 for detailed patient tumor characteristics. **(B)** Representative IF staining images of Schwann cell markers S100 (green) and Sox 10 (red) in SCH-2 cells, DAPI (blue). **(C)** Representative H&E staining of sciatic nerve SCH-2 tumors demonstrating classic schwannoma histology features of Verocay body (arrow pointed). Mice bearing SCH-2 tumors in the sciatic nerve model was evaluated for **(D)** the total duration of hind paw lifting behavior and **(E)** isolated licking behavior, in 5 min. **(F)** In mice receiving Sham surgery (Sham), or bearing SCH-1 and SCH-2 tumor, the motor function was evaluated by the rotarod test. In mice receiving Sham surgery (Sham), or bearing SCH-2 tumor, in the **(G)** sciatic nerve model and **(H)** spine model, the motor function was evaluated by the rotarod test at the indicated time post-implantation. The average time to fall from the rotating cylinder was normalized to the value from each mouse on the first day and presented as relative rotarod endurance. **(I)** von Frey filament test was performed in male or female mice receiving sham surgery or bearing SCH-2 tumors in the sciatic nerve. **P<0.005. All animal studies are presented as mean±SEM, n=8 mice/group, and are representative of at least three independent experiments.

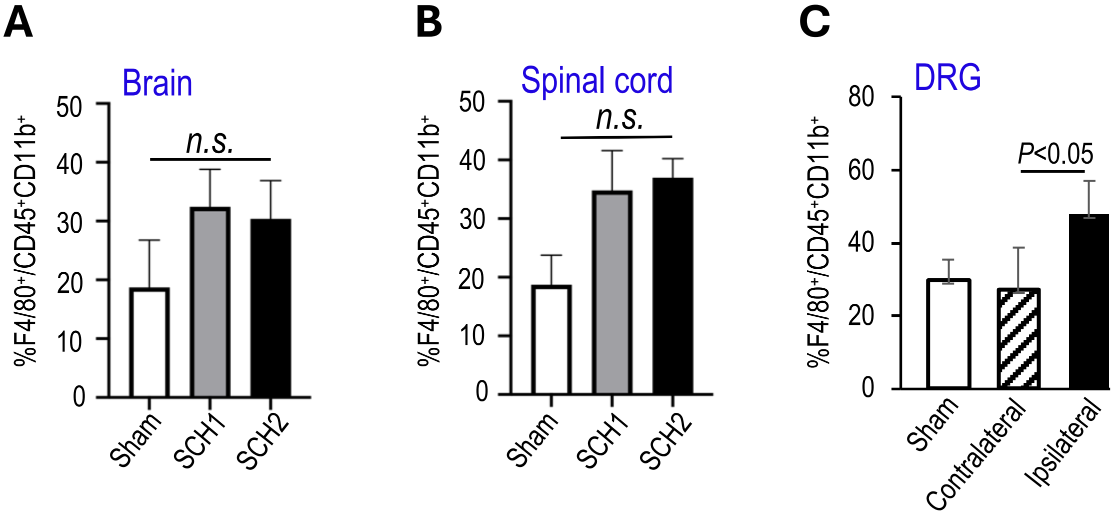

Figure S2. Evaluation of macrophage infiltration in the PDX model. (A) Flow cytometry analysis of the number of macrophages in the brain of mice in the sham, SCH-1, and SCH-2 groups. N=3 mice/group. **(B)** Flow cytometry analysis of the number of macrophages in the spinal cord of mice in the sham, SCH-1, and SCH-2 groups. N=3 mice/group. **(C)** Flow cytometry analysis of the number of macrophages in the ipsilateral and contralateral DRGs (L3-L6) in the sciatic nerve model of mice in the sham and SCH-2 groups. N=3 mice/group, 12 DRGs/group.

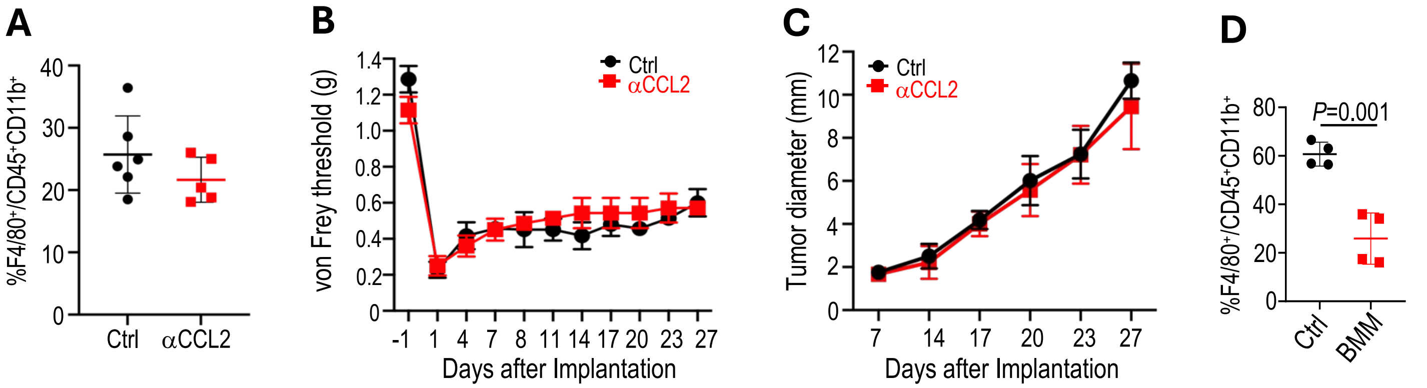

Figure S3. Macrophages in the DRG are essential in schwannoma-induced pain. (A) Flow cytometry analysis of the number of macrophages in lumbar DRGs (L3-L6) ipsilateral to sciatic nerve tumor in control and anti-CCL2-treated SCH-1 model. **(B)** Pain behavior was evaluated by the von Frey filament test at the indicated time points. **(C)** Tumor diameter was measured with a caliper. **(D)** Flow cytometry confirmed reduced macrophages in the adoptively transferred mice.

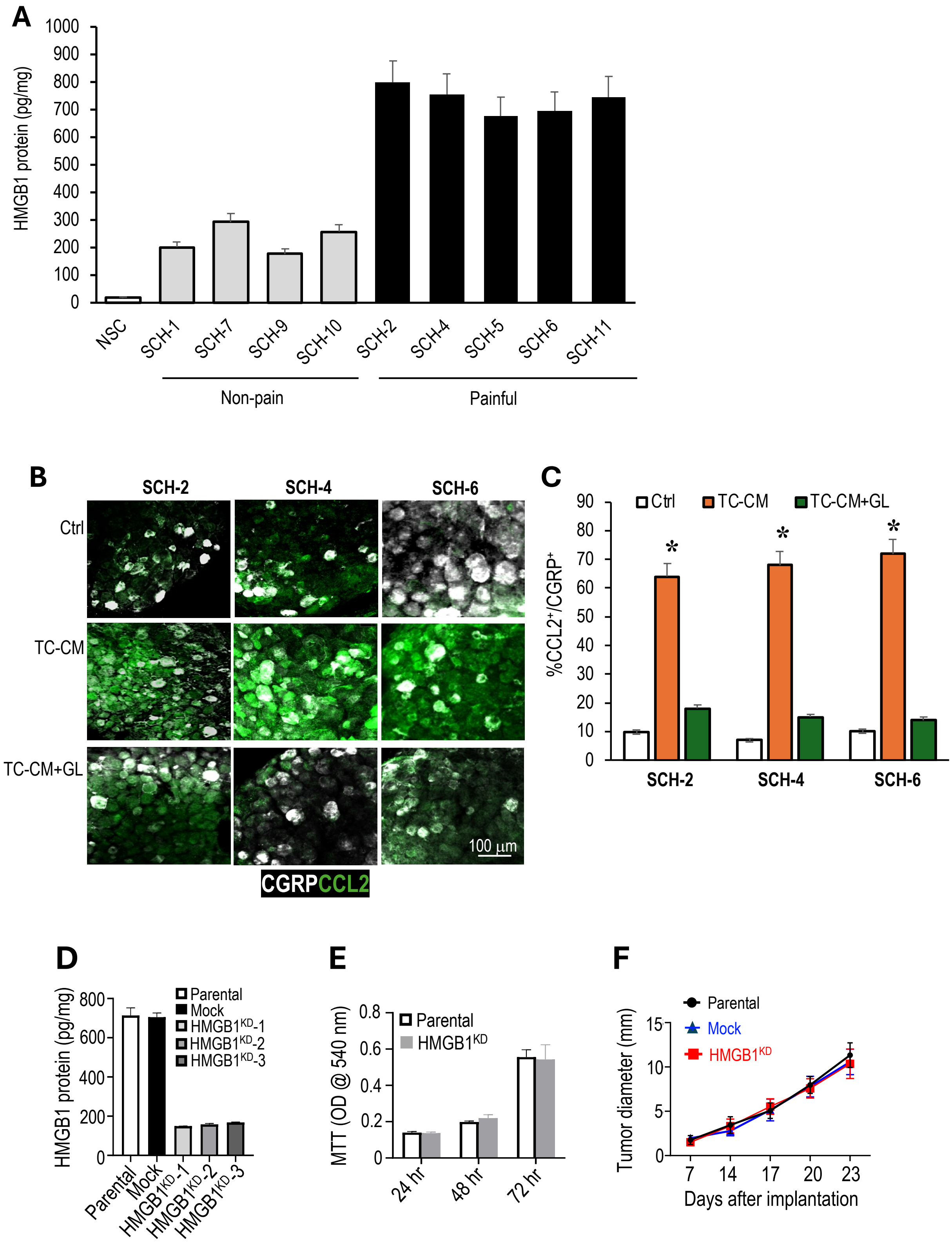

Figure S4. Characterization of HMGB1 expression in SWN cells and its functional role. (A) *In vitro* ELISA analysis of secreted HMGB1 protein in the supernatant of patient-derived schwannoma cells. **(B)** DRGs were organotypically cultured in DMEM (Ctrl), SCH-2, SCH-4, and SCH-6 tumor cell conditioned medium (TC-CM), or TC-CM+HMGB1 inhibitor, Glycyrrhizin (GL, 50 µg/ml) for 48 hours. Representative image of IF staining for CCL2 (green) in large-diameter ($CGRP^{+}$, red) neurons. **(C)** Image quantification of the percentage

of CCL2-expressing large neurons ($CGRP^+$) in 10 randomly selected fields (N=30 fields/group). **(D)** Confirmation of HMGB1 expression knockdown. HMGB1 protein levels in parental, mock-transfected, and 3 HMGB1 knockdown clones were measured by ELISA. **(E)** In vitro cell viability was assessed using the MTT assay. **(F)** Parental, mock-transfected, and HMGB1-knockdown cells were implanted into the sciatic nerve model. Tumor diameter was measured by caliper every 3 days. Animal studies are presented as mean±SEM, n=8 mice/group, and are representative of at least three independent experiments.

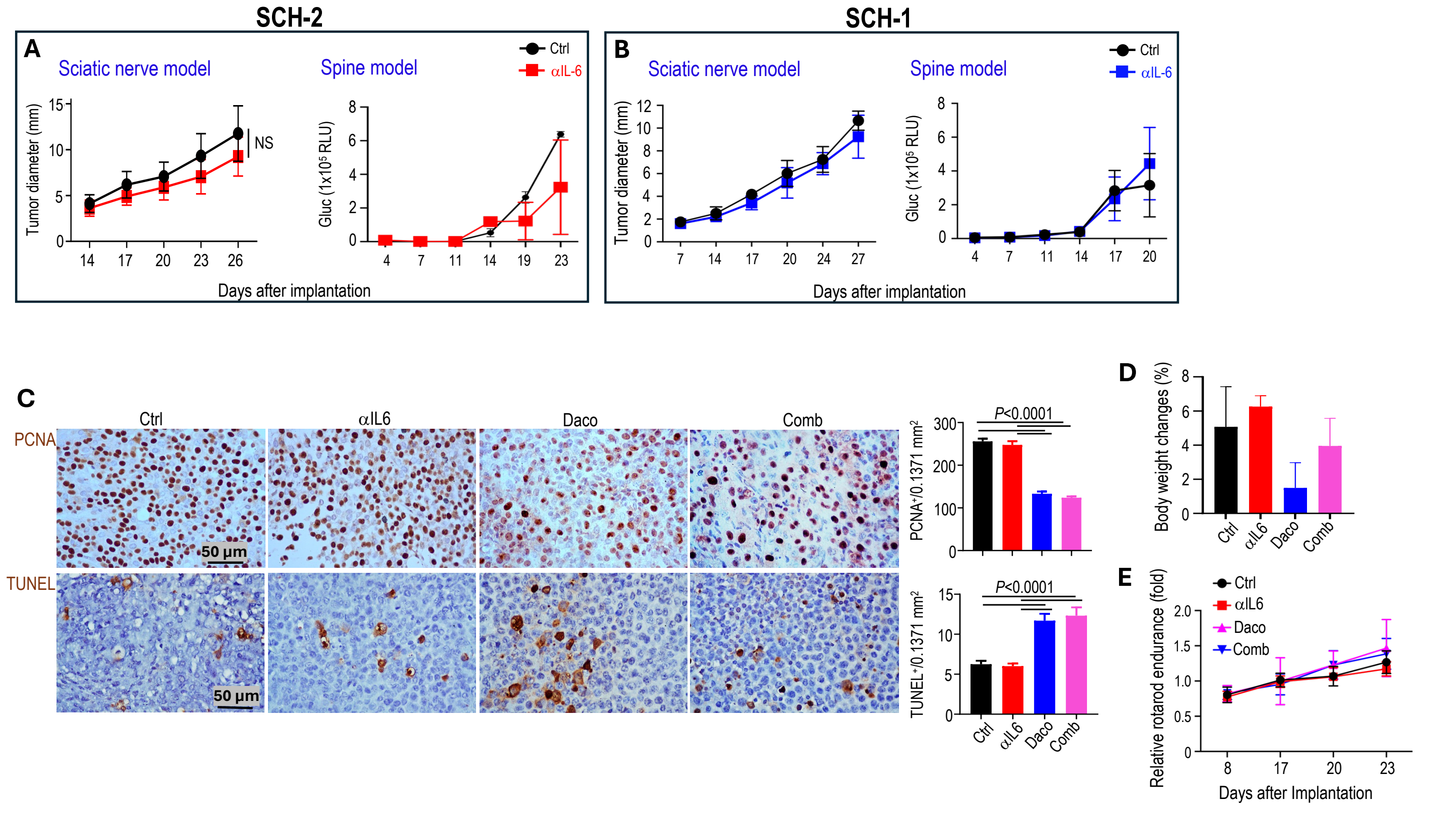

Figure S5. Combined IL-6 and EGFR blockade in vivo. (A-B) Anti-IL-6 treatment effect on tumor growth. **(A)** SCH-2 tumor growth in the sciatic nerve model was evaluated by measuring tumor diameter using a caliper, and tumor growth in the spine model was measured by blood Gluc level, at the indicated time. **(B)** SCH-1 tumor growth in the sciatic nerve model was evaluated by measuring tumor diameter using a caliper, and tumor growth in the spine model was measured by blood Gluc level, at the indicated time. **(C-E)** Combined IL-6 and EGFR blockade *in vivo*. **(C)** Representative IHC images of PCNA and TUNEL staining. Sciatic nerve tumors were collected at the experiment endpoints (when tumor size reached 1 cm in diameter) and stained for PCNA to mark proliferating cells and TUNEL to mark apoptotic cells. Quantification of positively stained cells was performed manually by counting 20 random areas (0.1371 mm^2). N=8 tumors/group. Data presents mean ± SD. **(D)** Treatment toxicity was evaluated by body weight changes monitored every 3 days. **(E)** Motor function was evaluated using the rotarod test. Data presented as mean±SEM, n=8 mice/group, and are representative of at least three independent experiments.
